# Construction of EGCG/chlorhexidine functionalized coating to reinforce the soft tissue seal at transmucosal region of implants

**DOI:** 10.1093/rb/rbaf046

**Published:** 2025-05-20

**Authors:** Lijie Zhang, Tiancheng Gao, Huaxue Qu, Bolin Li, Yuan Li, Yi Zhang, Tianxiang Dai, Tianshuo Zhu, Wei Li, Weibo Zhang, Jialong Chen, Xiangyang Li

**Affiliations:** Key Laboratory of Oral Diseases Research of Anhui Province, College & Hospital of Stomatology, Anhui Medical University, Hefei 230032, China; Key Laboratory of Oral Diseases Research of Anhui Province, College & Hospital of Stomatology, Anhui Medical University, Hefei 230032, China; Key Laboratory of Oral Diseases Research of Anhui Province, College & Hospital of Stomatology, Anhui Medical University, Hefei 230032, China; Key Laboratory of Oral Diseases Research of Anhui Province, College & Hospital of Stomatology, Anhui Medical University, Hefei 230032, China; Key Laboratory of Oral Diseases Research of Anhui Province, College & Hospital of Stomatology, Anhui Medical University, Hefei 230032, China; Key Laboratory of Oral Diseases Research of Anhui Province, College & Hospital of Stomatology, Anhui Medical University, Hefei 230032, China; Key Laboratory of Oral Diseases Research of Anhui Province, College & Hospital of Stomatology, Anhui Medical University, Hefei 230032, China; Key Laboratory of Oral Diseases Research of Anhui Province, College & Hospital of Stomatology, Anhui Medical University, Hefei 230032, China; Key Laboratory of Oral Diseases Research of Anhui Province, College & Hospital of Stomatology, Anhui Medical University, Hefei 230032, China; Key Laboratory of Oral Diseases Research of Anhui Province, College & Hospital of Stomatology, Anhui Medical University, Hefei 230032, China; Key Laboratory of Oral Diseases Research of Anhui Province, College & Hospital of Stomatology, Anhui Medical University, Hefei 230032, China; Key Laboratory of Oral Diseases Research of Anhui Province, College & Hospital of Stomatology, Anhui Medical University, Hefei 230032, China

**Keywords:** dental implant, STS, anti-inflammatory, antibacterial

## Abstract

Recent advancements in dental implant technology have provided more reliable and durable solutions for patients. Soft tissue seal (STS) is crucial for achieving implant stability, maintaining tissue health and promoting integration with surrounding soft and hard tissues. However, the STS around implants is fragile and susceptible to disruption by oral pathogens, particularly in patients with periodontitis or poor oral hygiene, leading to complications such as peri-implant mucositis and peri-implantitis. To promote STS formation, it is crucial to maintain the balance between bacterial and host cells while effectively managing inflammation. Although titanium-based implants exhibit biocompatibility, they lack inherent antibacterial and anti-inflammatory properties. To address these challenges, we developed a dual-function antibacterial and anti-inflammatory coating using chlorhexidine (CHX) and epigallocatechin gallate (EGCG). CHX effectively reduces bacterial adhesion but may inhibit fibroblast proliferation, while EGCG provides antioxidant and anti-inflammatory benefits. Three types of EGCG/CHX composite coatings were developed on titanium surfaces at different pH values. These coatings exhibited enhanced bacterial resistance, reduced inflammation and ROS scavenging capabilities, with higher pH levels further improving their performance. *In vivo* studies also confirmed that these coatings effectively prevented bacterial adhesion, mitigated inflammation and promoted STS formation, thereby holding significant promise for enhancing the long-term success of dental implants.

## Graphical abstract

**Figure:**
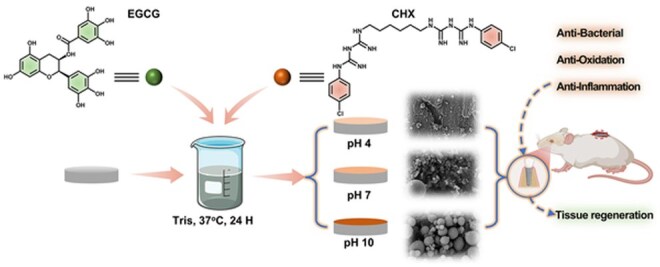

## Introduction

In recent years, dental implant technology has witnessed substantial advancements in the restoration of dental defects and missing teeth [1]. Despite the high retention and success rates associated with implants, failures do still occur [2]. In the past decades, a large number of studies have focused on improving osseointegration to enhance the therapeutic efficacy of implants [3, 4]. Recently, timely establishment and long-term maintenance of soft tissue sealing (STS) has garnered considerable attention due to its capacity to establish an effective transmucosal barrier against the invasion of various biological factors (either pathogens or inflammatory cytokines) [5], thereby protecting the underlying alveolar bone and ensuring the long-term function of the implant [6].

The establishment of STS relies on the attachment of gingival cells, including epithelial cells and fibroblasts [7]. However, pathological changes in the periodontal microenvironment of patients with periodontitis [8], poor oral hygiene or pre-existing conditions such as diabetes can impede the prompt establishment of STS, leading to a much higher implant failure rate compared to typical implant patients [9]. In these patients, the proportion and activity of pathogenic bacteria in the periodontal region are higher. These bacteria not only competitively adhere to the implant surface to block the attachment of gingival cells for STS establishment [10–12] but also secrete enzymes and toxins that destroy soft tissues [13]. Moreover, pathogenic bacteria can lead to the production of large amounts of reactive oxygen species (ROS) [14] and sustained inflammatory responses, which not only delay the healing of soft tissues in the transgingival area [15, 16], but also destroy the structure of the soft tissues [17, 18]. Similarly, hyperglycemia in diabetic patients can trigger the accumulation of ROS and enduring inflammatory reactions [19]. All these factors can affect the establishment and maintenance of STS. Therefore, preventing the adhesion of pathogenic bacteria to the implant surface, inhibiting bacterial activity around the implant, suppressing inflammation and clearing ROS are crucial for establishing a good STS and maintaining long-term stability.

Titanium-based materials are widely used in dental implants due to their excellent biocompatibility and mechanical properties [20]. However, their ability to address periodontal risk factors is limited. To enhance the therapeutic effect, especially for patients with periodontitis, inadequate oral hygiene or underlying conditions like diabetes, it is crucial to endow the transgingival surface of implants with antibacterial, antioxidant and anti-inflammatory properties. This aids in accelerating the establishment and improving the quality of the STS, ultimately enhancing treatment efficacy. The subgingival microbial environment is complex, and broad-spectrum antimicrobial agents like Ag^+^ and chlorhexidine (CHX) demonstrate superior therapeutic effects [21]. CHX, a cationic surfactant, disrupts bacterial membranes, causing them to break and release their contents. It is not susceptible to drug resistance, making it the gold standard for plaque control [22]. The grafting of 1% chlorhexidine acetate onto the implant surface effectively reduced the adhesion of various pathogenic bacteria on the implant surface [23], but CHX also inhibits the adhesion and proliferation of fibroblasts, potentially hindering the formation of STS [24, 25]. Additionally, the immobilization of CHX on the surface results in low loading of chlorhexidine and inability to release into the surrounding area to inhibit bacterial proliferation in the periodontal pocket [26].

EGCG exhibits potent antioxidant and anti-inflammatory properties, demonstrating therapeutic potential for inflammatory diseases [27]. However, its application is limited by low bioavailability and insufficient chemical stability [28]. Nanoparticles loaded with EGCG could achieve ROS clearance and induce macrophages to transform from pro-inflammatory M1 type to pro-repair M2 type, downregulate the expression of pro-inflammatory cytokines and remodel the inflammatory microenvironment [29, 30]. Moreover, EGCG is rich in negatively charged phenolic hydroxyl groups, which can interact with positively charged substances [31]. Consequently, extensive research has been conducted on using EGCG to develop various composite coatings, which plays a crucial role in the surface modification of biomaterials.

Here, we use the positive charge of CHX and the negative charge of EGCG, along with EGCG's metal chelation ability, to fabricate three types of EGCG/CHX composite coatings on the surface of smooth titanium. EGCG is a polyphenolic compound consisting of two catechin units linked by an ester bond, featuring hydroxyl (-OH) and gallate (-COOH) groups [32]. The interaction between EGCG and CHX varies with pH due to alterations in charge states, molecular structures and intermolecular interactions. Under acidic conditions (pH < 4), the hydroxyl groups of EGCG and the guanidyl group of CHX become protonated, thereby weakening electrostatic attraction and hydrogen bonding. This destabilizes EGCG's structure, leading to degradation [33]. At high pH levels (pH > 10), EGCG undergoes deprotonation, increasing its negative charge and causing structural degradation. Above pH 11, ring-opening reactions occur [34]. Therefore, solutions with pH values of 4, 7 and 10 were employed for the reaction. Biological performance evaluations demonstrated that the prepared coatings effectively prevented bacterial adhesion to the titanium surface, inhibited bacterial proliferation around the material, suppressed the secretion of pro-inflammatory cytokines and scavenged ROS. Furthermore, as the pH value of the reaction mixture increased, the antibacterial and anti-inflammatory properties of the prepared surfaces progressively improved, and they were longer effective. *In vivo* subcutaneous implantation studies revealed that the pH 7 and pH 10 coatings were effective in preventing bacterial adhesion and reducing the severity of inflammation.

## Materials and methods

### Materials

Commercial pure Ti was purchased from Baoji Non-ferrous Metal Co., Ltd. (Shanxi Province, China). Epigallocatechin gallate (EGCG) was purchased from Solarbio. Chlorhexidine (CHX) was purchased from Sigma-Aldrich. α-minimum Eagle’s medium (α-MEM), Fetal bovine serum (FBS), Trypsin-EDTA solution, brain–heart infusion (BHI) and LIVE/DEAD BacLight Bacterial Viability Kit™ (Invitrogen, Carlsbad, CA, USA) were purchased from Gibco. Cell Counting Kit-8 was purchased from Biosharp. Enzyme Linked Immunosorbent Assay Kit was purchased from Elabscience. RNA Extraction Kit was purchased from EScience Biotech.

### Preparation of EGCG/CHX composite coating

Commercial pure Ti disks were sequentially mechanically polished from 80 to 2000 grit, then ultrasonically cleaned with acetone, ethanol and deionized water, with each step repeated three times for 10 min, followed by drying and denoted as Ti. Deionized water with pH values of 4, 7 and 10 was used to dissolve EGCG and CHX powders, respectively. The two solutions were then mixed in equal volumes to prepare EGCG/CHX mixtures with different pH values (the concentrations of EGCG and CHX were 1.5 mg/mL and 1 mg/mL, respectively). Ti disks were immersed in these mixed solutions for 24 h, then, ultrasonically cleaned with deionized water for 5 min to obtain different EGCG/CHX coatings, denoted as EGCG/CHX 4, EGCG/CHX 7 and EGCG/CHX 10.

### Characterization of EGCG/CHX composite coating

#### Characterization of EGCG/CHX products

After 24 h of reaction, the EGCG/CHX mixtures were ultrasonically dispersed for 5 min, and then, centrifuged at 10 000 rpm for 5 min. The supernatant was discarded, and the precipitate was washed with deionized water, followed by centrifugation again. This process was repeated three times. Finally, after resuspending the precipitate in deionized water, a high-sensitivity Zeta potential and particle size analyzer (NanoBrook-90Pus PALS, Brookhaven) was employed to measure the zeta potential of the product, and a UV-Vis spectrophotometer (UV1800, Shimadzu) was employed to detect the absorption spectrum of the product at wavelengths ranging from 190 nm to 490 nm.

#### Characterization of EGCG/CHX coatings

After coating with gold for 30 s, the surface morphology of different samples was observed using scanning electron microscopy (SEM, Gemini 300, ZEISS). Surface roughness was detected by atomic force microscopy (AFM, NanoWizard 4XP, Bruker). The functional groups on the sample surfaces were detected by attenuated total reflection Fourier transform infrared spectroscopy (ATR-FTIR, Nicolet STIR20SX, Thermo Fisher Scientific). Surface chemical composition was analyzed by X-ray photoelectron spectroscopy (XPS, ESCALAB 250, Thermo) with a pass energy of 100 eV for wide-scans, with binding energy (BE) ranging from 0 to 1400 eV; at the same time, a pass energy of 30 eV was used to analyze the elemental states of different samples, obtaining high-resolution detailed scans. The system was calibrated using the C1s peak at 284.8 eV. Due to the take-off angle set at 45°, the detection depth does not exceed 10 nm. The collected data were quantitatively analyzed and curve-fitted using the XPspeak software package. Water contact angles were measured with deionized water at room temperature to study the wettability of different surfaces by contact angle meter (WCA, OCA 15EC, Dataphysics).

Specifically, under acidic conditions (pH = 2), the ultraviolet absorption spectra of the two components displayed distinguishable characteristic peaks (Figure S3A–C). Notably, at a wavelength of 313 nm, CHX showed no absorption, whereas EGCG demonstrated pronounced absorption. Leveraging this distinct difference in spectral characteristics, the study employed ultraviolet dual-wavelength spectrophotometry. The wavelengths of 313 nm (specific to EGCG) and 252 nm (characteristic of CHX) were selected for quantitative analysis, and standard curves for both compounds were subsequently established (Figure S3D–F). The samples were stored in a 37°C dark environment (to prevent photodegradation of EGCG) and immersed in deionized water for 1, 3, 5, 7, 9, 11 and 14 days, respectively. At each time point, the coating was dissolved using 1 mL of acidic deionized water (pH = 2). To ensure complete dissolution, as verified by X-ray photoelectron spectroscopy (XPS), the samples underwent 24 h of oscillation followed by 10 min of ultrasonic treatment. Subsequently, the absorbance was measured at dual wavelengths (313 nm and 252 nm) using ultraviolet–visible spectrophotometry. The concentrations of EGCG and CHX were determined via standard curves, and cumulative release profiles over time were plotted. Five parallel sample sets were prepared, with blank deionized water serving as a control to ensure data reliability. This approach effectively minimized spectral interference and enabled precise quantification of the two active components. The release amounts were quantified by measuring the total EGCG and CHX content on the sample surface under pH = 2 conditions. The release levels at different time points were calculated by subtracting the remaining EGCG and CHX content on the sample surface after immersion from the initial EGCG and CHX content on the un-immersed sample.

#### Antioxidant activity of EGCG/CHX coatings

The ROS scavenging efficiency of different samples was assessed by DPPH assay and ABTS assay. The sample was immersed in 1 mL of DPPH ethanol solution with a concentration of 2 mM, incubated at 37°C for 6 hr in the dark, then absorbed 150 μl of solution, measured the absorbance at 517 nm, and obtained the radical scavenging effect by the following formula. Besides, an aqueous solution of ABTS with a concentration of 7.4 mM was mixed with an equal volume of aqueous solution of potassium persulfate with a concentration of 2.6 mM, and then, the mixture was stored in the dark at room temperature for 12 h to obtain the ABTS radical cation (ABTS^+^) solution. This radical solution was diluted with PBS to an absorbance of 0.70 ± 0.10 measured at 734 nm to obtain a working solution. The sample was immersed in 1 mL of working solution, incubated at 37°C for 30 min in the dark, then absorbed 150 μl of solution, measured the absorbance at 734 nm, and obtained the radical scavenging effect by the following formula.


$$\text{Scavenging effect}=\text{1}00\times ({\text{Absorbance}}_{\text{blank}}-{\text{Absorbance}}_{\text{sample}})/{\text{Absorbance}}_{\text{blank}}$$

### Antibacterial assessment

The antibacterial evaluation in this study includes the assessment of the material's surface ability to inhibit bacterial adhesion and the material's ability to suppress bacterial proliferation in the surrounding environment of *Staphylococcus aureus (S. a)*, *A. actinomycetemcomitans (A.a)* and *Porphyromonas gingivalis (P. g)*. *S. a* was grown at 37°C for 24 h in brain heart infusion (BHI) broth for 24 h, *P. gingivalis* was cultured in BHI broth supplemented with hemin (5 μg/mL) and Vitamin K (10 μg/mL) in an anaerobic atmosphere of 80% N_2_, 10% H_2_ and 10% CO_2_ for 48 h, and *A.a* was cultured in BHI broth supplemented with L-cysteine (0.2 mg/mL) and 0.5% yeast extract (5 mg/mL) in anaerobic atmosphere for 48 h. The concentration of bacterial suspension was diluted to 10^6^ CFU/ml for the following antibacterial evaluations.

#### Inhibition of bacterial adhesion on material surfaces

After the UV-sterilized samples were placed on 24-well plates, 60 μl of bacterial suspension was added to the surface of each sample and incubated at 37°C for 4 h. Then, 1 mL of BHI broth was added to each well for a certain period of time (24 h for *S. a*, and 48 h for *A.a* and *P. g*). A portion of the samples were removed, rinsed with deionized water, and then, placed into a centrifuge tube containing 1 mL of physiological saline. Sonication for 1 min and vortexing for 3 min were applied to elute the bacteria from the sample surface. Subsequently, 100 μl of eluent from each tube was evenly spread onto solid BHI agar plates (for *S. a*) or blood agar plates (for *A.a* and *P. g*) for further incubation, and photographs of the bacteria on the plates were taken. Another portion of the samples was rinsed with physiological saline, stained with the LIVE/DEAD^®^ BacLight™ bacterial viability kit for 15 min, and then, observed under a fluorescence upright microscope (DM600B, Leica) to assess the live and dead bacteria on the sample surface. The final portion of the samples was taken out, rinsed and sequentially dehydrated in a gradient of ethanol solutions (25%, 50%, 75%, 100% for 15 min step by step), dealcoholization, critical point dried, gold coated and finally, the bacteria on the sample’s surface were observed using SEM.

#### Inhibiting bacterial proliferation around materials

Forty microliter of bacterial suspension was evenly spread on solid BHI agar plates (for *S. aureus*) or blood agar plates (for *A. a* and *P. gingivalis*). Then, one part of sterilized samples was placed face down on the agar surface and incubated at 37°C for a certain period, then the zones of inhibition (ZOI) around the samples was recorded by photograph.

#### Antibacterial evaluation of subcutaneous implanted samples in vivo

The samples were implanted into the subcutaneous area of SD rats to evaluate the antibacterial ability of the samples *in vivo*. After anesthetizing SD rats, the posterior implantation area was shaved and the skin was incised longitudinally to create a 10 mm incision to expose the superficial layer of the deep fascia; subsequently, subcutaneous tissues were gently separated using blunt dissection to form a soft tissue cavity; then, 60 μl of *S. aureus* suspension was injected and the samples were placed into the soft tissue cavity; finally, the subcutaneous tissue and skin were sutured with surgical thread. On the first- and seventh-days postoperation, the rats were euthanized, the samples were removed and pressed face down on a solid agar plate for 1 min, then, the samples was removed and the solid agar plate was further cultured for 24 h to observe bacterial proliferation. Another portion of the samples was placed into a centrifuge tube containing 1 mL of physiological saline, sonicated for 1 min to elute the bacteria from the sample surface. Subsequently, 100 μl of eluent was evenly spread onto solid agar plates, cultured at 37°C for 24 h and the bacterial growth was observed.

### Cytocompatibility assessment

The mouse fibroblasts (L929) were used to assess the cell compatibility of the samples. Cells were cultured with alpha-Minimum Eagle’s medium (α-MEM) supplemented with 10% fetal bovine serum under 37°C, 5% CO_2_ conditions. The sterilized samples were placed in 24-well plates, and 1 mL cell suspension with a density of 5 × 10^4^ cells/ml was added to each well, and the medium was changed every 2 days. After incubation for 1, 3 and 5 days, some samples were tested for cell proliferation by cell counting kit-8. The other sample was rinsed with normal saline and fixed with 2.5% glutaraldehyde for 4 h, then, stained with Rhodamine 123 for 15 min and observed under fluorescence microscope.

### Co-culture of bacteria-cells

To observe the effect of the sample on the competitive adhesion of bacteria and cells on the sample surface, a co-culture experiment with bacteria and cells was conducted. Sterilized samples were placed in a 24-well plate, with 60 μl of *S. aureus* suspension added to the surface of each sample, and incubated at 37°C for 4 h. Then, 20 μl of cell suspension of fibroblast at a concentration of 5 × 10^5^ cells/ml was added to the sample surface, and after incubation for 15 min, 1 mL of cell suspension at a concentration of 1 × 10^4^ cells/ml was added to each well. After 1 day and 3 days of culture at 37°C and 5% CO_2_, the samples were removed and rinsed with physiological saline, then stained with LIVE/DEAD^®^ BacLight™ bacterial viability kit (Invitrogen, America) for 15 min, and the number of live/dead bacteria and cells on the sample surface was observed by fluorescence microscopy.

### Anti-inflammatory effect of samples

#### Intracellular ROS elimination

The mouse fibroblasts (L929) and mouse mononuclear macrophage leukemia cells (RAW 264.7) were used to assess the intracellular ROS elimination cell compatibility of the samples. Cells were cultured with alpha-Minimum Eagle’s medium (α-MEM) supplemented with 10% fetal bovine serum (FBS) under 37°C, 5% CO_2_ conditions. The samples were immersed in 1.5 mL of α-MEM medium with 10% FBS and incubated at 37°C for 24 h. The resulting medium was collected as the extract for the following tests.

Initially, 1 mL of a fibroblast suspension at a density of 2 × 10^4^ cells/well was added to each well and incubated for an additional 24 h. Following this, the medium was aspirated, and 1 mL of the eluent containing 300 μM H_2_O_2_ was introduced into the wells for a 6-hr culture period. After the extract was removed, 1 mL of serum-free medium containing 10 μM of the ROS-specific probe DCFH-DA (MedChemExpress, America)was added and the cells were cultured in the dark for 30 min. Subsequently, the medium was decanted, and the cells were rinsed three times with serum-free medium. Finally, fluorescence and bright-field images of the cells were captured using an inverted fluorescence microscope (Axio Observer 3, ZEISS).

Furthermore, 1 mL of a RAW264.7 cell suspension at a density of 1 × 10^5^ cells/well was added to a 24-well plate for a 24-h culture. After the medium was gently aspirated, 1 mL of the eluent containing 300 μM H_2_O_2_ was added for a 6-hr culture. Subsequently, the medium was gently aspirated, and 1 mL of serum-free medium containing 10 μM ROS-specific probe DCFH-DA was added and incubated in the dark at 37°C for 30 min. The cells were then dislodged from the plate bottom by vigorous pipetting and collected into a 1.5 mL Eppendorf tube. After centrifugation to discard the supernatant, the cell pellet was washed with serum-free medium three times. Finally, fluorescence and bright-field images of the cells were captured using an inverted fluorescence microscope, meanwhiles, flow cytometry (FACSCelesta, BD Biosciences) was employed to determine the percentage of oxidant-stressed RAW 264.7 cells in each sample group.

#### Relative mRNA expression of inflammatory factors and cytokine secretion of macrophage

To evaluate the effect of the coatings on inflammation, sterilized sample surfaces were inoculated with 60 μl of a *P. gingivalis* suspension at a density of 1 × 10^6^ CFU/ml, and incubated for 1 h. Subsequently, 1 mL of sterile culture medium was added, and the incubation was continued for an additional 24 h. The culture medium was then collected, and 500 μl of the supernatant, devoid of bacteria, was centrifuged at 5000 rpm for 10 min and set aside for subsequent use. In a 12-well plate, 2 mL of macrophage suspension at a density of 3 × 10^5^ cells/ml was added to each well and cultured for 24 h. Following this, the aforementioned supernatant was added and the culture was extended for another 24 h. The cells were resuspended by pipetting, and the culture medium was transferred to centrifuge tubes. The cells collected in centrifuge tubes were centrifuged at 3000 rpm, after which the supernatant was collected for proinflammatory cytokine quantification, while the cell pellet was processed for RNA extraction followed by quantitative real-time PCR (qRT-PCR) analysis. The cell pellet was washed with 1 mL of PBS, centrifuged again and the cells were collected. RNA was extracted according to a rapid RNA extraction protocol, followed by reverse transcription and sample preparation. The expression levels of mRNA associated with pro-inflammatory cytokines (TNF-α, IL-6 and IL-1β) were analyzed and compared based on the CT values obtained. Additionally, the supernatant was centrifuged at 4°C and 12 000 rpm for 20 min to remove cell debris. The supernatant was then collected and the amount of pro-inflammatory cytokines secreted by the cells was measured according to the methods provided in the kit.

#### Evaluation of subcutaneous inflammatory response in vivo

To evaluate the effect of the sample on inflammation in a bacterial environment *in vivo*, the sample is implanted subcutaneously in a contaminated area of SD rats. After anesthetizing SD rats, the skin of the posterior implantation area was incised longitudinally to create a 10 mm incision and subcutaneous tissues were gently separated using blunt dissection to form a soft tissue cavity. Then, 60 μl of *S. aureus* suspension was injected and the samples were placed into the soft tissue cavity; finally, the subcutaneous tissue and skin were sutured with surgical thread. After four weeks of implantation, some rats were euthanized by excessive anesthesia and the samples were removed from the body. The soft tissue surrounding the samples is separated, fixed with a 4% paraformaldehyde solution for about 7 days, paraffin-embedded, sectioned and stained with hematoxylin and eosin (HE). Finally, the TG panoramic tissue cell quantitative analysis system (TissueFAXS Plus S, TissueGnostics) is used to capture images of the fibrous capsule surrounding the samples *in vivo* for inflammation analysis.

To further evaluate the soft tissue sealing efficacy of the samples in the oral cavity, a peri-implantitis model was established in the oral cavity of rats. The bilateral maxillary first molars were extracted, and the coated implants were randomly implanted into the extraction sockets. *P. gingivalis* was inoculated around the implants throughout the experimental period. At week 4, the rats were euthanized, and the peri-implant soft tissues were carefully isolated under sterile conditions. The tissues were washed with pre-cooled phosphate-buffered saline (PBS) to remove residual blood. Hundred milligram of soft tissue were weighed and minced. The minced tissue was transferred to a tissue grinding tube containing 900 μl of PBS supplemented with protease inhibitors. Homogenization was performed using a tissue grinder set to skin grinding mode at 4°C, 70 Hz for 10 min. After centrifugation at 5000 rpm for 10 min, the supernatant was carefully collected. The concentrations of proinflammatory cytokines TNF-α, IL-6 and IL-1β in the supernatants were subsequently quantified using rat-specific ELISA kits (Elabscience, China). The remaining maxillary samples were fixed in 4% paraformaldehyde solution for 7 days, decalcified at low temperature for 1 month, embedded in paraffin, sectioned, stained and imaged using the TG Panoramic tissue cell quantitative analysis system for inflammation analysis. All animal tests were performed following the protocols accessed by the Laboratory Animal Administration Rules of China and Local Ethical Committee (LLSC-20231193).

### Statistics

All experimental data were obtained from three independent replicates and are presented as mean ± standard deviation (Mean ± SD). Comparisons between two groups were analyzed using Student’s *t*-test, while comparisons among three or four groups were assessed by one-way analysis of variance (One-Way ANOVA). Statistical significance was defined as follows: *P* < 0.05 (*), *P* < 0.01 (**), *P* < 0.001 (***) and ‘ns’ indicates no statistical significance.

## Results and discussion

### Surface physic-chemical characterization

**Figure 1.**
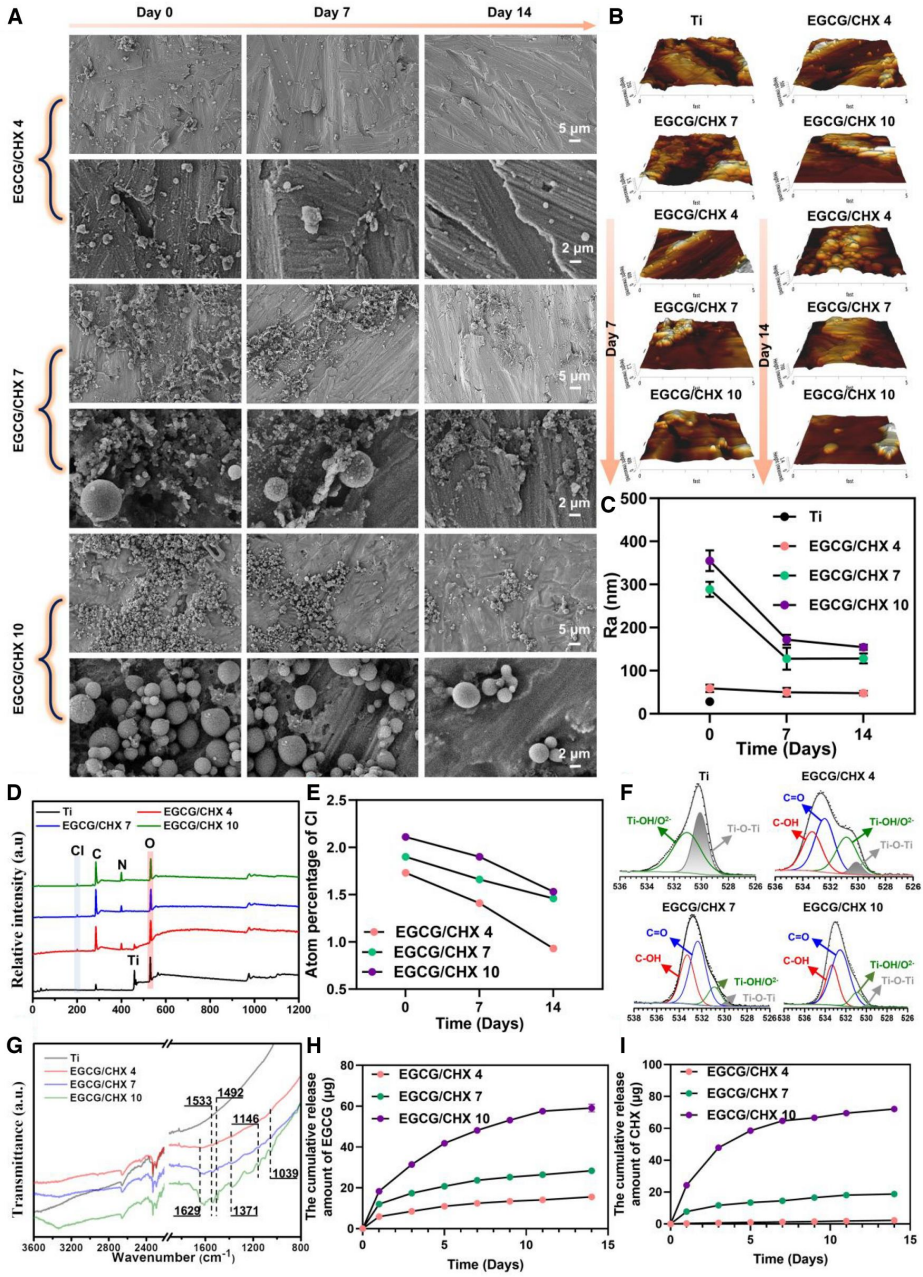
(**A**) SEM images of the surfaces of samples from different groups; (**B**) AFM characterization of coating morphology on titanium sheet surfaces; (**C**) the surface roughness of the sample; (**D**) wide-scan XPS spectra; (**E**) atomic percentage analysis; (**F**) O1s peak fittings of different pH values; (**G**) FTIR analysis; (**H**) the release curve of EGCG; (**I**) the release curve of CHX.

SEM and AFM were used to investigate the surface morphology and roughness of the samples, respectively, as shown in (Figure 1A–C and Figure S2). The surface of titanium is relatively uniform, with an average roughness (*Ra*) of 28.2 nm. Meanwhile, deposits were observed on the surfaces of the EGCG/CHX samples in all three groups, and the amount of deposits on the surfaces and Ra value increased with the increase of solution pH value. The EGCG/CHX4 surface is scattered with a small number of irregular particles, leading to an increase in *Ra* to 58.8 nm. The EGCG/CHX7 surface exhibits a large amount of deposits, among which a small portion are spherical particles with diameters ranging from 200 to 600 nm, and the *Ra* increases to 288.6 nm. The EGCG/CHX10 surface also shows a significant amount of deposits, with the vast majority being spherical nanoparticles with diameters between 80 and 400 nm, and the Ra increases to 354.9 nm. These results indicate the successful deposition of EGCG and CHX on the titanium surface.

To evaluate the stability of the coatings, samples were immersed in deionized water for 7 and 14 days, and the changes of surface morphology and average roughness were observed. The results show that after 7 days of immersion, the EGCG/CHX 4 surface only has sporadic deposits, with the *Ra* value decreasing to 49.7 nm, while the deposits on the EGCG/CHX 7 and EGCG/CHX 10 surfaces are significantly reduced in size and quantity, but there are still a large number of deposits on both surfaces, which lead to the *Ra* value decreasing to 127.6 nm for EGCG/CHX 7 and 171.7 nm for EGCG/CHX 10. After 14 days of immersion, the EGCG/CHX 4 surface shows little change with the *Ra* value slightly decreasing to 47.7 nm. The deposits on the EGCG/CHX 7 surface are further reduced, leaving only irregular deposits and with Ra value about 128.2 nm. The spherical particles deposit on the EGCG/CHX 10 surface are also further reduced in quantity and size, leading to a decrease in *Ra* to 154.2 nm. These results demonstrate that the EGCG/CHX coatings will gradually release and maintain their presence for over two weeks.

XPS analysis was conducted to investigate the atom ratio and surface chemical composition of different surfaces. The wide-scan XPS spectra and atomic percentage are displayed in (Figure 1D and E and Table 1), respectively. The intensity of the Ti 2p peak on the prepared EGCG/CHX surfaces was significantly lower than that on the Ti surface, particularly with the Ti 2p peak disappearing on surfaces of EGCG/CHX 7 and EGCG/CHX 10. Additionally, the atomic percentage (at%) of Ti on the surfaces was 19.99% for Ti, 2.66% for EGCG/CHX 4, 0.25% for EGCG/CHX 7 and 0.25% for EGCG/CHX 10. The decrease in titanium content confirms the successful construction of the EGCG/CHX coatings, and the surfaces of EGCG/CHX 7 and EGCG/CHX 10 have more deposits than the EGCG/CHX 4 surface, which is consistent with the SEM results. Compared to Ti, the intensity of the N 1 s peak on the prepared surfaces strengthened, and a Cl 2p peak appeared. The atomic percentage of N and Cl was 1.73% and 8.78% for EGCG/CHX 4, 1.9% and 10.97% for EGCG/CHX 7 and 2.11% and 11.2% for EGCG/CHX 10, respectively. Because N and Cl are only present in chlorhexidine (C_22_H_30_Cl_2_N_10_), these results prove that chlorhexidine has successfully integrated to the sample surfaces. To demonstrate the binding of EGCG on the material surface, O1s peak fittings were further performed (Figure 1F and Table 2), and the detailed information is shown in Table 2. Compared to that in the high-resolution spectrum of Ti, two peaks in the O1s spectrum of the three EGCG/CHX surfaces both appear at 532.4 eV and 533.4 eV, which correspond to the C = O and C-OH of EGCG, respectively, indicating that EGCG is integrated to these surfaces. Among the three EGCG/CHX surfaces, the area percentage of C = O in O1s and C-OH in O1s both increase with the increase of the solution pH, proving that the amount of EGCG on these surfaces gradually increases.

**Table 1.** Surface chemical elemental composition of sample (atomic %) was obtained by XPS

	*Cl*	*C*	*N*	*Ti*	*O*
Ti	0.24	24.89	2.00	19.99	52.88
EGCG/CHX 4	1.73	62.99	8.78	2.66	23.83
EGCG/CHX 4-7d	1.41	59.01	9.42	3.01	27.15
EGCG/CHX 4-14d	0.93	53.97	7.22	5.92	31.96
EGCG/CHX 7	1.9	63.3	10.97	0.25	23.58
EGCG/CHX 7-7d	1.66	61.84	9.74	2.73	24.03
EGCG/CHX 7-14d	1.46	60.52	8.65	2.51	26.87
EGCG/CHX 10	2.11	62.84	11.20	0.25	23.61
EGCG/CHX 10-7d	1.90	64.51	12.73	0.44	20.42
EGCG/CHX 10-14d	1.53	63.79	12.74	0.53	21.40

**Table 2.** Binding energies (eV) and area percentages (%) of the deconvoluted XPS O1s peaks

Binding energy (eV)		530	∼530.8	∼532.4	∼533.4
Peak assignment		Ti-O-Ti	Ti-OH/C-O	C = O	C-OH
Area percentage of peak (%)	Ti	49.8	50.2	0	0
	EGCG/CHX 4	5.2	29.5	32	33.3
	EGCG/CHX 4-7d	15	39.7	27.5	17.8
	EGCG/CHX 4-14d	18.6	40.5	26.1	14.8
	EGCG/CHX 7	1.4	11	46.3	41.3
	EGCG/CHX 7-7d	3.1	27.1	41.7	28.1
	EGCG/CHX 7-14d	8.7	35.8	39.6	15.9
	EGCG/CHX 10	0.5	5.9	48.6	45
	EGCG/CHX 10-7d	3.7	23.2	45	28.1
	EGCG/CHX 10-14d	5.1	26.3	44	24.6

XPS was also used to measure the elemental composition on the surfaces of released samples to evaluate the stability of the coatings. After the samples were immersed for 7 and 14 days, the atomic percentage of N and Cl on the EGCG/CHX surfaces gradually increased with immersion time, indicating that CHX was gradually released from these surfaces. Additionally, the atomic percentage of Ti also increased with the increase of immersion time, suggesting that more of the substrate was exposed, also proving that the coating was gradually released. However, even after 14 days immersion, Cl2p was still present on these surfaces, indicating that CHX was not completely released. Meanwhile, the O1s peak of these surfaces fitting was carried out. The results showed that the proportion of C = O and C-OH in O1s on the three released EGCG/CHX surfaces gradually decreased with extended immersion time, but the area percentage of these two peaks remained relatively large, also proving that the coating can be maintained for more than 14 days.

To investigate the interaction of EGCG and CHX, ATR-FTIR analysis was performed. As shown in (Figure 1G), compared with Ti, the EGCG/CHX coatings have a wide peak at 3300–3400 cm^−1^ corresponding to NH and -OH. The peaks at 1629, 1533 and 1492 cm^−1^ correspond to the aromatic group C = O, and the peaks at 1146 and 1039 cm^−1^ correspond to C-O, which proves that EGCG is successfully integrated to the titanium surface. The peak at 1371 cm^−1^ corresponding to C-N proves that CHX is successfully integrated to the titanium surface. In addition, these peak intensities increased with the increase of solution pH, indicating that the amount of EGCG and CHX on the surface increased.

To measure the amount of EGCG and CHX on the surface of these samples, samples were immersed in a pH 2 sulfuric acid solution to completely separate ECGC and CHX from the surfaces, and then, the absorbance of these solution was measured by UV–Vis spectrophotometer and the amount of each substance was calculated by the dual wavelength method. XPS detected the surface of dissolved samples and found that Cl and N elements disappeared, proving that sulfuric acid can completely dissolve the surface deposits. As shown in Figure S3G and H, with the increase of pH value, the amount of the EGCG/CHX coating increased, and the proportion of CHX in the coating gradually increased. The amounts of CHX and EGCG on the surfaces of the released samples were also measured. The amounts of both components on the surfaces decreased rapidly with increasing immersion time. After 14 days of immersion, the residual rates of EGCG and CHX on the surfaces were 11.6% and 70.4% for EGCG/CHX 4, 23.4% and 37.7% for EGCG/CHX 7, 13% and 28.7% for EGCG/CHX 10, respectively, which also demonstrated that these coatings were not completely released after 14 days of immersion. Furthermore, as illustrated in Figure 1H and I, which depict the EGCG and CHX release curves for each coating group, the release rates of both EGCG and CHX significantly decelerated after 7 days of immersion. Notably, the cumulative release amount of EGCG during the immersion period exceeded that of CHX. In conjunction with the assessment of the antibacterial efficacy of the released samples (Figure S5), to some extent, the variations in the antibacterial properties among the groups after release can, to some extent, be attributed to the influence of chlorhexidine on the antibacterial effect during the release process. Additionally, by integrating the subsequent anti-inflammatory and antioxidant effects, a more comprehensive evaluation of the biological impacts of EGCG's release behavior is achieved.

The variation in surface hydrophilicity can indirectly reflect changes in surface properties, hence, the water contact angle (WCA) was measured, with the results shown in Figure S1. The WCA values are 43.22 ± 0.71^°^ for Ti, 74.34 ± 0.98^°^ for EGCG/CHX 4, 80.63 ± 0.94^°^ for EGCG/CHX 7 and 84.90 ± 0.38^°^ for EGCG/CHX 10, respectively. The significant increase in WCA of the EGCG/CHX coatings indicates that the coatings were successfully constructed on the surface. Studies have shown that surfaces grafted with CHX have a water contact angle of 40–50° [25], Surfaces modified with tannic acid (TA), a polyphenolic compound structurally similar to EGCG, have a water contact angle of 30–50° [22], but when combined with CHX, the water contact angle increases dramatically, possibly due to the exposure of more hydrophobic groups. To investigate the reactivity of EGCG and CHX in solutions with different pH values, the UV–Vis absorption intensity of the obtained precipitates was measured. As shown in Figure S3, the absorbance values of the reaction product with 10 times dilution increased significantly with the increase of the reaction solution pH, proving that the reactivity and yield of the two substances increase with pH. The stronger the reactivity of EGCG and CHX, the more they combine on the material surface. Additionally, the ζ-potential of the reaction products was measured, as shown in Figure S4. The products obtained from acidic reaction solutions have a large positive charge, while the products obtained from neutral and alkaline reaction solutions have only a small positive charge. The greater the charge on the product, the greater the repulsive force between them, making it difficult for them to aggregate, and preventing them from overcoming gravity to come into contact with the titanium surface. The products obtained in neutral and alkaline reaction solutions have a small positive charge, which makes them easy to aggregate and deposit, thereby increasing the likelihood of contact with the titanium surface. Studies have shown that the titanium surface has a slight positive charge (6.43 mV) [22]. Thus, products with a lower positive charge are more capable of overcoming electrostatic repulsion forces compared to those with higher positive charges, facilitating their interaction with the titanium surface. The presence of EGCG in the product enhances metal chelation capabilities. Upon contact with the titanium surface, the negatively charged EGCG will form strong bond with titanium through the chelating metal ability and its negative charge. The hydrophilicity of the surfaces after immersion was also measured, and the results show that as the immersion time increases, the surface hydrophilicity gradually improves, but the values of WCA are still much higher than that of Ti, also proving that these coatings were not fully released.

### Antibacterial assessment

After implantation, bacterial colonization on the implant surface can impede the adhesion of STS-related cells. Pathogenic bacteria in the peri-implant environment can either prevent the establishment of STS or compromise existing STS structures by secreting substances such as lipopolysaccharides and endotoxins [35] or by inducing excessive inflammatory responses [36]. Therefore, *S.aureus (S.a), A.actinomycetemcomitans (A.a)* and *P.gingivalis (P.g)* were used to evaluate antibacterial property of these samples. SEM images and Live/dead bacterial staining were used to evaluate the number and status of adhesive bacteria on the material surface. Bacterial plating and counting results further validate the antibacterial ability of these coatings. The inhibition zone is utilized to assess the antibacterial efficacy of each sample against surrounding microorganisms.

**Figure 2.**
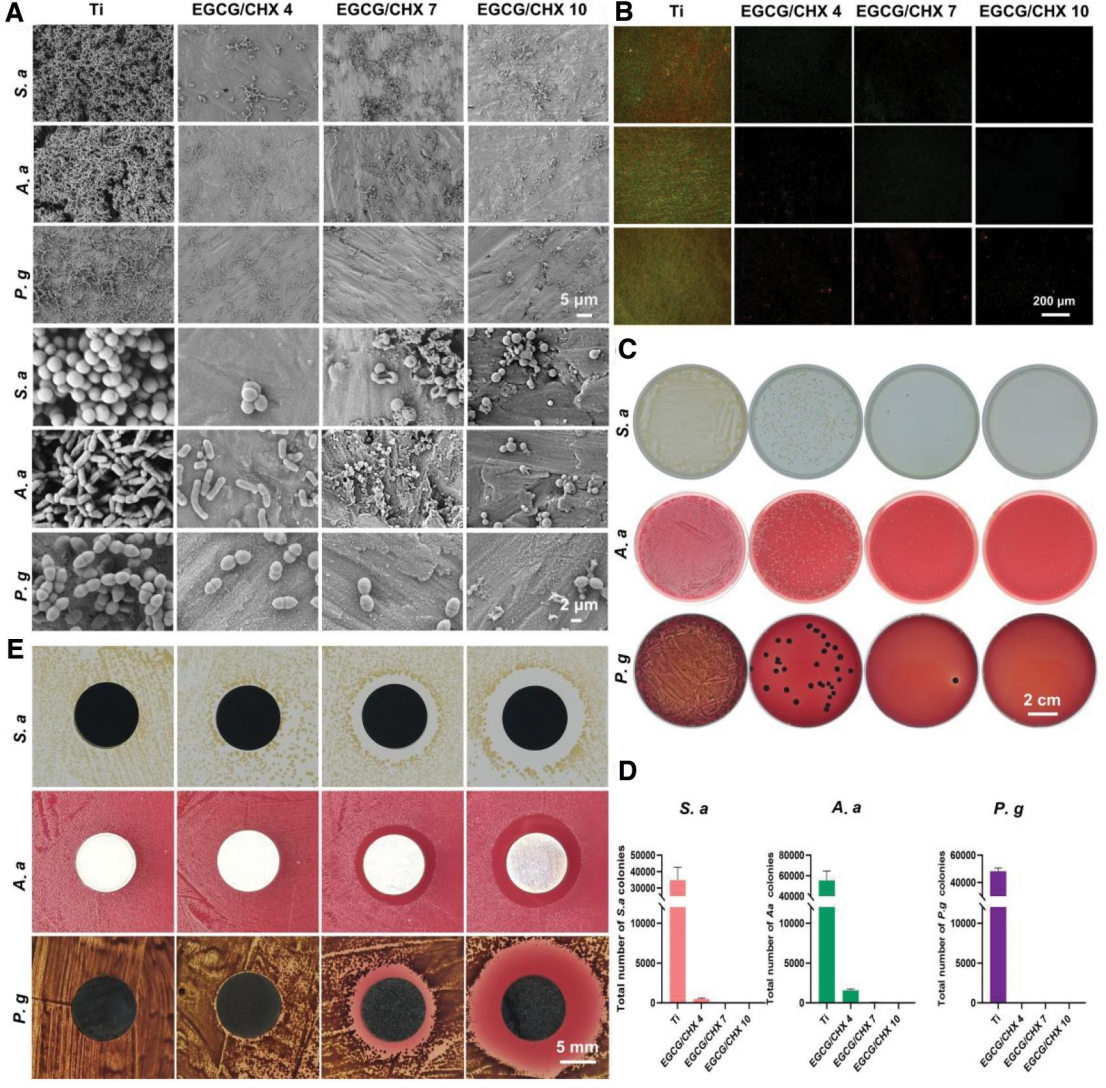
Antibacterial effect on the surface of sample before release. (**A**) SEM observation of surface bacteria on samples with different components; (**B**) liquid antibacterial bacterial plate culture results; (**C**) and counting results; (**D**) different groups of samples antibacterial zones; (**E**) staining of live/dead bacteria results.

SEM results in Figure 2A show that the titanium surface is completely covered with bacteria, exhibiting multi-layered accumulation. While the EGCG/CHX 4 surface has a smaller number of bacteria, and the EGCG/CHX 7 and EGCG/CHX 10 surfaces only have sporadic bacteria present. The viability of adherent bacteria on the surface was evaluated using a live/dead bacterial staining. Live bacteria are green in color, dead bacteria are red and bacteria in apoptosis are yellow. As shown in Figure 2B, the Ti surface is completely covered with a large number of bacteria, with the dead bacteria possibly resulting from natural death due to overpopulation and the deficiency of nutrients and space [37, 38]. The number of bacteria adhering to the EGCG/CHX 4, EGCG/CHX 7 and EGCG/CHX 10 surfaces is significantly lower than that on the Ti surface, with the number decreasing in sequence and the proportion of dead bacteria increasing successively. The bacteria on the sample surfaces were eluted and spread-plated to determine the number of viable bacteria on the surfaces. As shown in Figure 2C, the photographs of the spread plates indicate that there are a large number of bacterial colonies in the titanium group, while the number of bacterial colonies in the EGCG/CHX-modified groups is significantly reduced, especially in the EGCG/CHX 7 and EGCG/CHX 10 groups, where only a few scattered and sporadic colonies are observed. The above results were statistically analyzed through colony counting (Figure 2D), and the conclusion was the same as above that the EGCG/CHX-modified samples effectively inhibit bacterial adhesion on the material surface, with the samples of EGCG/CHX 7 and EGCG/CHX 10 demonstrating a strong bactericidal effect on adherent bacteria.

The ability of the samples to inhibit bacteria in the surrounding environment was evaluated using the ZOI method, which primarily measures the antibacterial substances released by the samples into the surrounding environment to suppress the growth of bacteria around the material, forming a sterile zone. As shown in Figure 2E, there was no clear sterile area around Ti, while there were circular inhibition zones around the EGCG/CHX samples, and the widths of the inhibition zones were 0.59 mm for EGCG/CHX 4, 1.97 mm for EGCG/CHX 7 and 3.15 mm for EGCG/CHX 10 in the *S.a* group, 0 mm for EGCG/CHX 4, 1.65 mm for EGCG/CHX 7 and 2.63 mm for EGCG/CHX 10 in the *A.a* group, and 0.51 mm for EGCG/CHX 4, 1.98 mm for EGCG/CHX 7 and 5.7 mm for EGCG/CHX 10 in the *P.g* group, indicating that the inhibitory effect of the EGCG/CHX samples on bacteria in the surrounding environment increases with the pH value of the preparation solutions.

**Figure 3.**
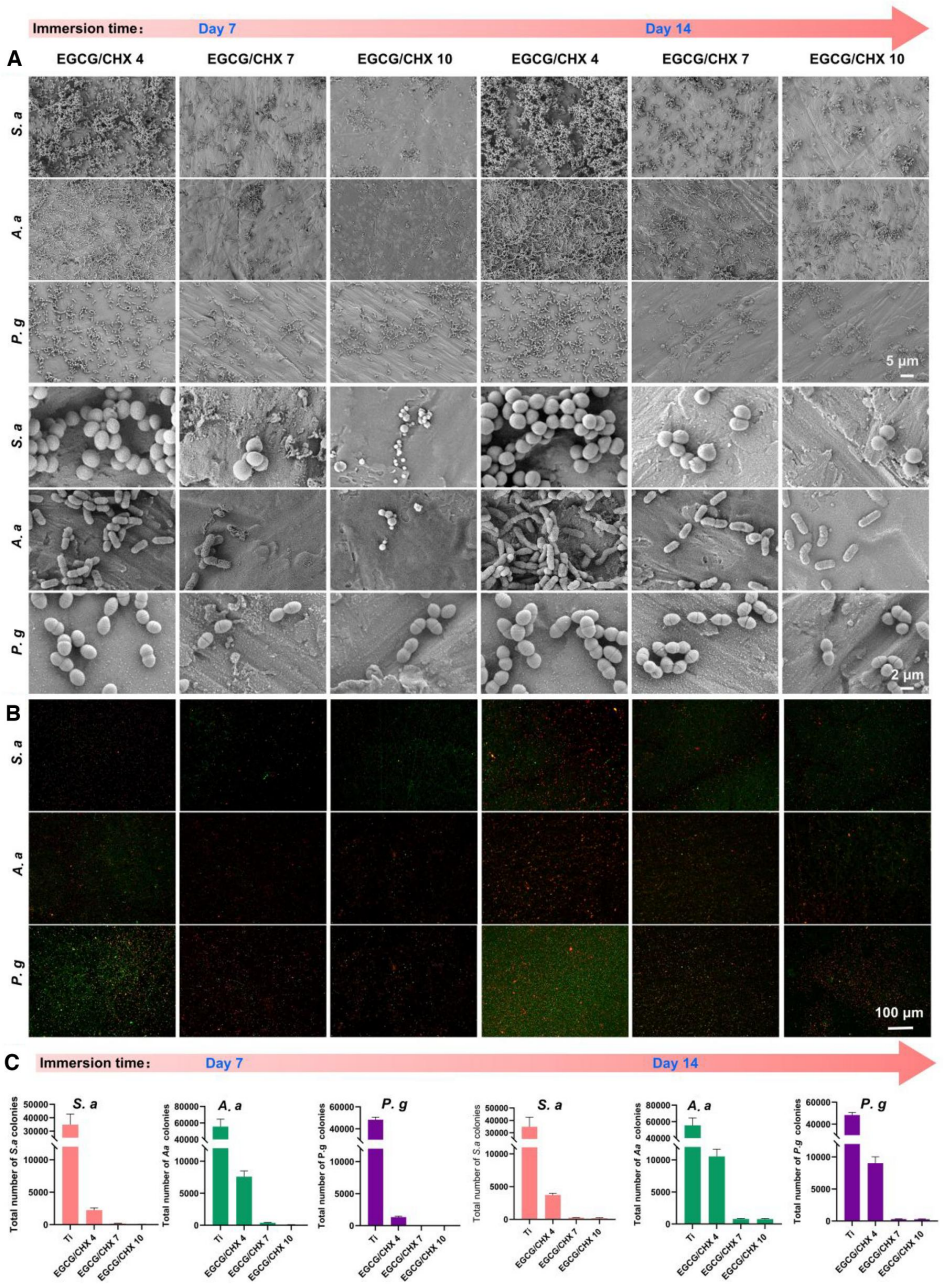
Antibacterial effect on the surface of sample after release. (**A**) SEM observation of surface bacteria on samples with different components; (**B**) staining of live/dead bacteria results; (**C**) liquid antibacterial bacterial plate culture counting results of samples after release.

The bacterial adhesion on the surface of released EGCG/CHX samples was also evaluated by SEM observation (Figure 3A), live/dead bacterial staining (Figure 3B) spread plate count (Figure 3C). The number of bacteria on the released EGCG/CHX surfaces increased significantly with the extension of the release time. However, the number of adhesive bacteria on these surfaces was still lower than that on the Ti surface. The EGCG/CHX 7 and EGCG/CHX 10 surfaces still had very few bacteria, with a high proportion of dead bacteria, proving that the antibacterial ability of these surfaces can be maintained for at least 14 days, especially the surface of EGCG/CHX 10. The ability of the released samples to inhibit bacteria in the surrounding environment was evaluated using the ZOI method as shown in Figure S5. As the immersion time of the samples increased, the width of the inhibition zones decreased. In the *S.a* and *P.g* groups, the EGCG/CHX 7 and EGCG/CHX 10 samples still had sterile zones, proving that the released chlorhexidine from these samples can still inhibit the proliferation of these two types of bacteria, while in the *A.a* group, no inhibition zones were found in all the released EGCG/CHX samples, proving that the chlorhexidine released from these samples was not enough to inhibit the proliferation of *A.a*.

### Cytocompatibility test

**Figure 4.**
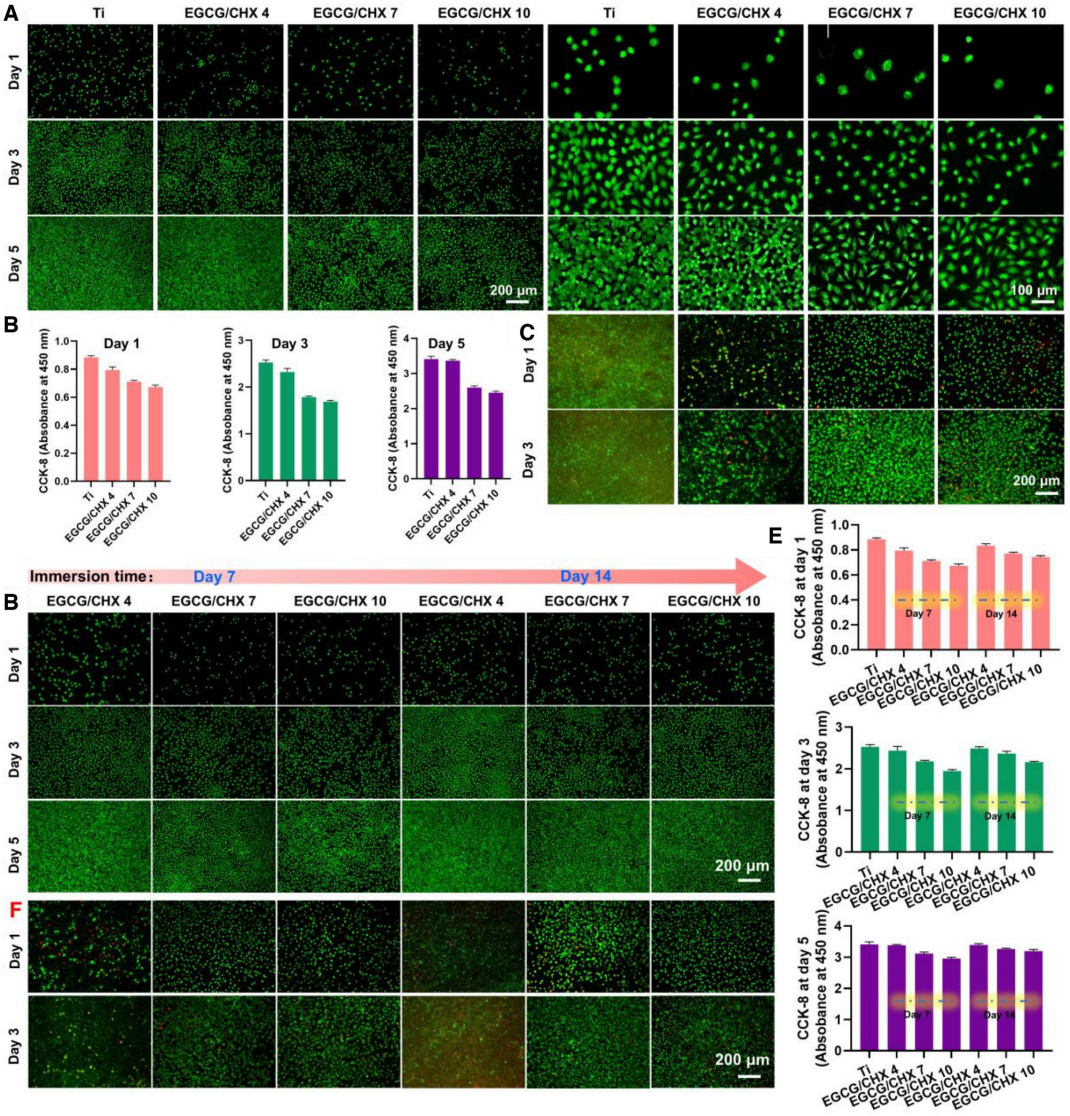
Adhesion and proliferation of fibroblasts on different groups of sample surfaces. (**A**) Fluorescence staining results; (**B**) CCK-8 results; (**C**) bacterial/cell co-culture (L929/*S. a*) on sample surface; (**D**) fluorescence staining results of surface cells after sample release; (**E**) bacterial/cell co-culture on surface after sample release. (**F**) CCK-8 results of cells after sample release.

The STS is established by the robust adhesion of junctional epithelial cells and fibroblasts, however, surfaces modified with antimicrobial agents typically exhibit cytotoxicity [39]. Therefore, L929 were employed to evaluate the cytocompatibility of the materials. The adhesion and proliferation of fibroblasts on the surfaces were studied by fluorescence staining and CCK-8 assay, with results presented in Figure 4A and B. One day post-inoculation, the surfaces exhibited a descending order of adherent cell count as follows: Ti > EGCG/CHX 4 > EGCG/CHX 7 > EGCG/CHX 10, with cell viability obtained by CCK-8 on EGCG/CHX 4, EGCG/CHX 7 and EGCG/CHX 10 surfaces being 89.7%, 80.7% and 76.1% of that on the Ti surface, respectively. Till day 5, the cell viability on EGCG/CHX 4, EGCG/CHX 7 and EGCG/CHX 10 surfaces were 98.5%, 76.2% and 71.8% of that on the Ti surface, respectively. The cell viability on all sample surfaces had multiplied to 3.88 times for Ti, 4.25 times for EGCG/CHX4, 3.66 times for EGCG/CHX 7 and 3.66 times for EGCG/CHX 10 by day 5 compared to day 1. These results showed that the surface cytocompatibility of EGCG/CHX 7 and EGCG/CHX 10 was slightly poor, but EGCG/CHX modification did not inhibit cell proliferation.

In clinical, the transmucosal region of dental implants is an open area, hence the attachment of STS-related cells on the implant surface is inevitably subjected to bacterial interference, especially for patients with poor periodontal health and diabetes [16, 40, 41]. Therefore, the adhesion and proliferation of cells in a bacterial environment have clinical guidance value [42]. *S. aureus*, as one of the pathogenic bacteria to adhere to the material surface, was co-cultured with fibroblasts to evaluate the material's clinical performance. As shown in Figure 4C, when the bacteria/cells were co-cultured for 1 day, the Ti surface was completely covered by bacteria, whereas only a small number of bacteria and cells adhered to the EGCG/CHX 4 surface, with a significant proportion of dead cells. In contrast, the EGCG/CHX 7 and EGCG/CHX 10 surfaces were predominantly populated by live cells and a minimal number of dead cells, with no bacteria detected. After 3 days of co-culturing, the bacteria on the Ti surface formed a denser film, and the proportion of red dead bacteria increased, speculated due to overpopulation and the deficiency of nutrients and space. In contrast, the EGCG/CHX 7 and EGCG/CHX 10 surfaces showed a further increase in cell numbers with normal morphology, and again, no bacteria were detected. These results demonstrate that excellent antibacterial surfaces can ensure effective cell adhesion, speculated that EGCG/CHX 7 and EGCG/CHX 10 surfaces may perform more effectively *in vivo*, providing a more favorable environment for cell attachment and growth while inhibiting bacterial colonization.

The cytocompatibility of the released EGCG/CHX samples was also evaluated by fluorescence staining and CCK-8 assay, with results shown in Figure 4D and E. The results indicated that at three different culture time points, the cell number and viability of the released EGCG/CHX samples decreased with increasing pH value of the preparation solutions, yet remained higher than the non-released group and lower than the Ti group. Additionally, the longer the EGCG/CHX samples were released, the greater the increase in cell number and viability on these surfaces, with the most significant increases observed in EGCG/CHX 7 and EGCG/CHX 10. By day 5, the cell activity on the surfaces of EGCG/CHX 4-14d, EGCG/CHX 7-14d and EGCG/CHX 10–14 d was 99.4%, 95.6% and 93.5% of that on the Ti surface, respectively. These results demonstrate that the cytocompatibility of EGCG/CHX samples significantly improved with the release of the coating. It is speculated that this improvement is related to the gradual reduction of antimicrobial agents on the material surfaces.

Bacterial/cell co-culture was also employed to assess the cellular behavior of the released EGCG/CHX samples within an environment mimicking the bacterial challenges of periodontal tissues. Figure 4F illustrates that the EGCG/CHX 4-7d and EGCG/CHX 4-14d surfaces exhibited a marked increase in bacterial colonization compared to the unreleased samples (EGCG/CHX 4), with complete bacterial coverage observed on both surfaces by the third day of co-culture. In contrast, the EGCG/CHX 7-7d and EGCG/CHX 10-7d surfaces demonstrated an elevated cell adhesion compared to their unreleased samples, with no bacterial presence detected. Similarly, the EGCG/CHX 7-14d and EGCG/CHX 10-14d surfaces showed an increased cell adhesion, yet occasional bacterial colonies emerged, and the cells were unable to spread evenly, implying a potential detrimental impact of bacteria on cellular health. The above results suggest that the EGCG/CHX 7 and EGCG/CHX 10 coatings foster cell adhesion amidst bacterial contamination, with enhanced adhesion effects correlating with the release of the coating. Nonetheless, as the antibacterial properties diminish, the proliferation of bacteria could compromise cellular integrity and health.

### Anti-inflammatory of EGCG/CHX coatings

**Figure 5.**
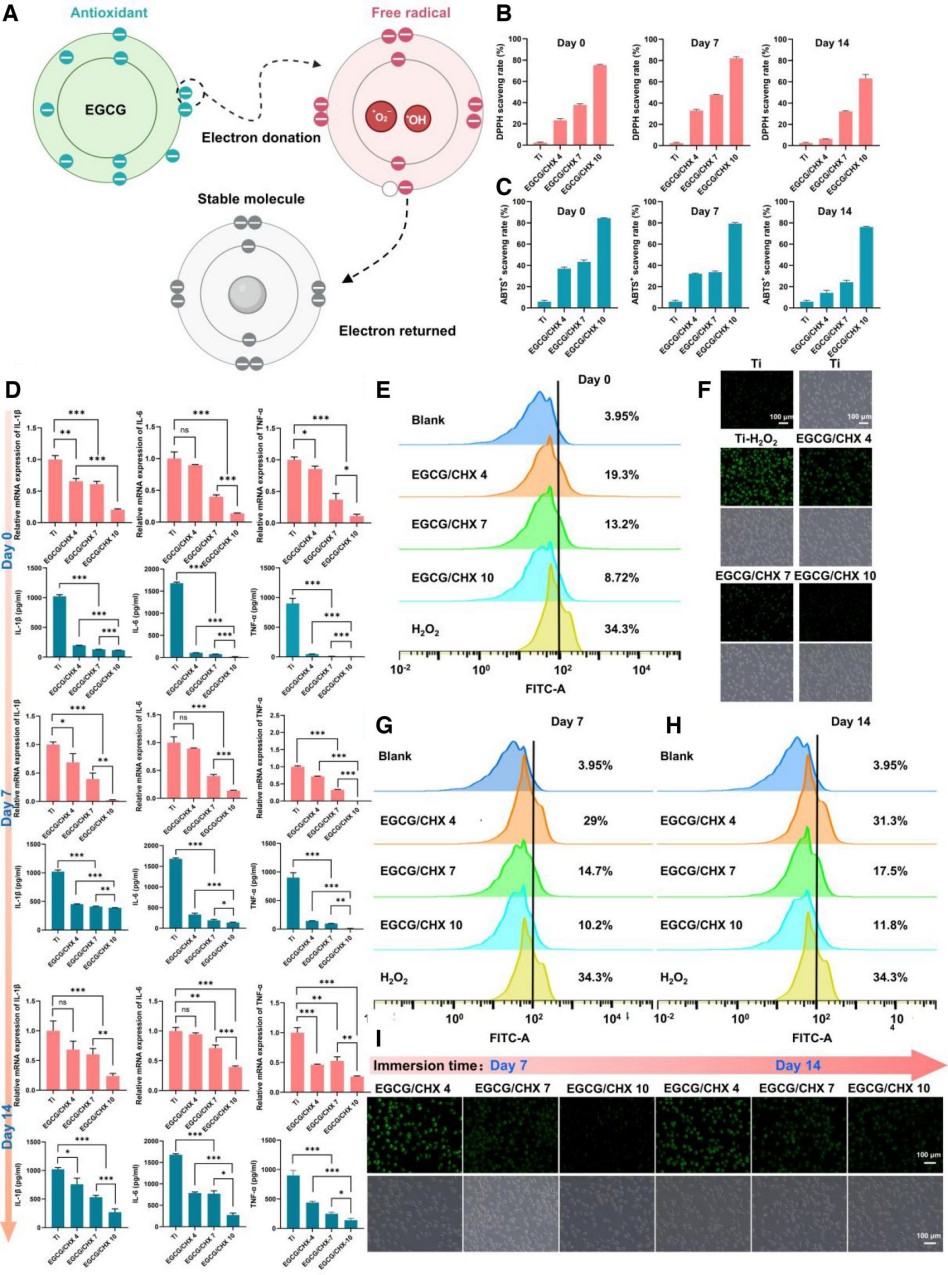
The antioxidant effect on the surface of samples from different groups. (**A**) EGCG mechanism for scavenging free radicals; (**B**) the effect of samples on scavenging DPPH free radicals; (**C**) and scavenging ABTS^+^ free radicals; (**D**) RT-qPCR and Elisa determination of pro-inflammatory cytokine levels; (**E**) flow cytometry data of macrophages; (**F**) fluorescence staining results of fibroblasts; (**G**) data of flow cytometry for detection of reactive oxygen species in macrophages 7 days after sample release; (**H**) data of flow cytometry for detection of reactive oxygen species in macrophages 14 days after sample release; (**I**) and fluorescence staining results of fibroblasts after sample release.

ROS can react with proteins, lipids and nucleic acids, resulting in oxidative stress and damage of these macromolecules, which can lead to diseases such as diabetes, periodontitis and cancer [43] and is also one of the key factors leading to the destruction of periodontal tissue. The ABTS^+^ and DPPH inactivation capacity of these samples was used to evaluate the material's free radical scavenging capacity (antioxidant capacity). Compared with the Ti, the three EGCG/CHX modified surfaces showed excellent scavenging capacity for both types of free radicals (Figure 5B and C), with the scavenging ability increasing with the pH value of the prepared samples and decreasing with the duration of immersion. Among them, the free radical scavenging ability of EGCG/CHX 10 samples which were not immersed and immersed for 14 days was much stronger than that of the other two groups, which is presumed to be related to the higher amount of EGCG on their surfaces, given that EGCG is known for its superior antioxidant properties.

The process of implantation can result in damage to the transgingival area, and the open environment of this region is prone to bacterial invasion, triggering a severe pro-inflammatory M1 polarization of macrophages and overexpression of pro-inflammatory cytokines, including tumor necrosis factor (TNF-α), interleukin (IL-6) and interleukin (IL-1β), which affect the formation of the STS [44]. To evaluate the sample's impact on inflammation within a bacterial environment, macrophages were cultured in medium that had been pre-incubated with the sample and bacteria. Subsequently, macrophages and culture medium were collected to determine the quantity of pro-inflammatory factor mRNA in the macrophages using RT-qPCR and the levels of pro-inflammatory cytokines secreted into the medium using ELISA (Figure 5D). The results indicated that the inflammatory response in the three EGCG/CHX sample groups was significantly lower than in the Ti group, and the expression of pro-inflammatory cytokines and related mRNA in these groups decreased significantly with increasing pH, demonstrating that the EGCG/CHX samples possess a strong ability to inhibit inflammation, with the inhibitory effect intensifying as the pH increases.

There are high levels of ROS in the transmucosal region of dental implants, especially for patients with poor periodontal health and diabetes, which can escalate cellular oxidative stress, prolong the pro-inflammatory state and impair cellular activity, thereby affecting the quality of STS [16]. Therefore, DCFH-DA fluorescence assay was employed to evaluate the inhibitory impact of EGCG/CHX coating eluates on ROS generation in macrophages RAW264.7 and L929 under H_2_O_2_-induced oxidative stress. The flow cytometry data of macrophages are shown in Figure 5E and the fluorescence staining of fibroblasts results are shown in Figure 5F. Compared with the Ti group without H_2_O_2_ induction, there was a significant increase in intracellular ROS levels of cell on the Ti induced by H_2_O_2_. However, within the three EGCG/CHX groups induced by H_2_O_2_ oxidative stress, a significantly reduced ROS production was observed in both cell types compared with the pure Ti group induced by H_2_O_2_, and the intracellular ROS production in the three groups was inversely correlated with pH. These results demonstrate that EGCG/CHX can effectively inhibit ROS production in fibroblasts and macrophages, thereby alleviating the adverse effects of oxidative damage, with the alleviation effect increasing as the pH value of the preparation solution, speculated to be related to the release of EGCG in the coating. The inhibitory effects of EGCG/CHX samples released for 7 days and 14 days on oxidative stress were analyzed. The quantitative analysis of ROS in macrophages was conducted using flow cytometry, and the results are shown in Figure 5G and H. The fluorescence staining results of fibroblasts are presented in Figure 5I. The results indicated that the intracellular ROS levels in the released EGCG/CHX sample groups remained lower than those in the pure titanium group induced by H_2_O_2_, but higher than the non-released sample group. Moreover, the ROS levels increased with extended release time and decreased with increasing pH values. These results demonstrate that as the EGCG/CHX coating gradually degrades, its antioxidant stress capability diminishes. However, the EGCG/CHX 7 and EGCG/CHX 10 groups can maintain this effect for at least 14 days.

### *In vivo* implantation experiment

**Figure 6.**
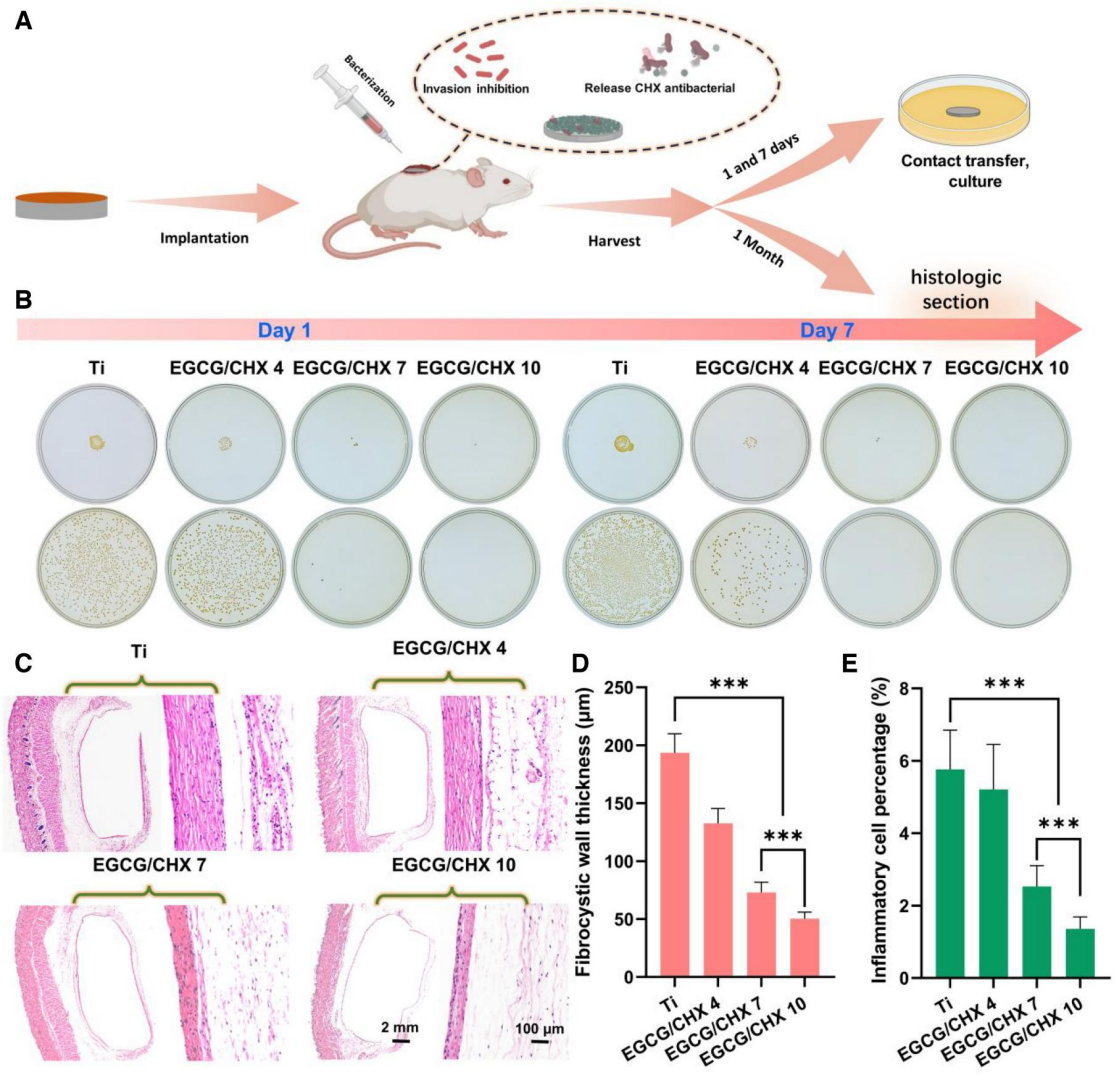
*In vivo* evaluation of back implants in rats. (**A**) Subcutaneous implantation model in rats to evaluate *in vivo* antibacterial and anti-inflammatory properties: modified materials were implanted subcutaneously followed by bacterial injection to simulate clinical infection; implants were harvested on days 1 and 7 for bacterial quantification using contact and spread plate methods, and tissue samples were collected at 1 month for histological analysis. (**B**) Press plate and spread-plate results of rat subcutaneous implants after removal 1 day and 7 days. (**C**) HE staining of the fibrous capsule surrounding the materials. (**D**) Thickness measurement of the fibrous capsule. (**E**) The proportion of inflammatory cells in the fibrous capsule.

To further verify the antibacterial efficacy of the samples *in vivo*, we established a *S. aureus* infection model in the subcutaneous soft tissue of the dorsal region of SD rats. After implantation for 1 day and 7 days, the samples were removed. Some samples were directly pressed onto agar plates for continued incubation for 24 h, while the bacteria on the surface of the remaining samples were eluted and then spread-plated for culture for 24 h, as shown in Figure 6A and B. The results revealed that after 1 day of implantation, substantial bacterial colonies were observed on the samples Ti and EGCG/CHX 4, while sparse colonies appeared on EGCG/CHX 7, and no bacterial colonies appeared on EGCG/CHX 10. After 7 days of implantation, a slight increment in colony count was observed for Ti, a modest decrease for EGCG/CHX 4, a further reduction for EGCG/CHX 7 and a continued absence of colonies for EGCG/CHX 10. These results suggest that as the pH value of the preparation solution increases, the antibacterial ability of the EGCG/CHX samples *in vivo* also increases. Moreover, as the implantation time extends, the antibacterial effect of these samples in the local area is enhanced. It is speculated that this is related to the amount of CHX loaded on the samples and the amount of CHX released.

To evaluate the impact of materials on soft tissue inflammation in a bacterial environment, materials were implanted into the subcutaneous tissue of rats with bacterial contamination. One month after implantation, the materials were removed and the surrounding tissues were separated for histological examination by HE staining (Figure 6C). The thickness of the fibrous capsule surrounding the samples was measured as an indicator of soft tissue inflammation. The results indicated thicknesses of 193.4 ± 16.5 μm for Ti, 132.6 ± 12.8 μm for EGCG/CHX 4, 73.0 ± 8.9 μm for EGCG/CHX 7 and 50.3 ± 5.7 μm for EGCG/CHX 10 (Figure 6D). Additionally, the area percentage of inflammatory cells in the capsule was 5.8% for Ti, 5.2% for EGCG/CHX 4, 2.5% for EGCG/CHX 7 and 1.4% for EGCG/CHX 10 (Figure 6E).

**Figure 7.**
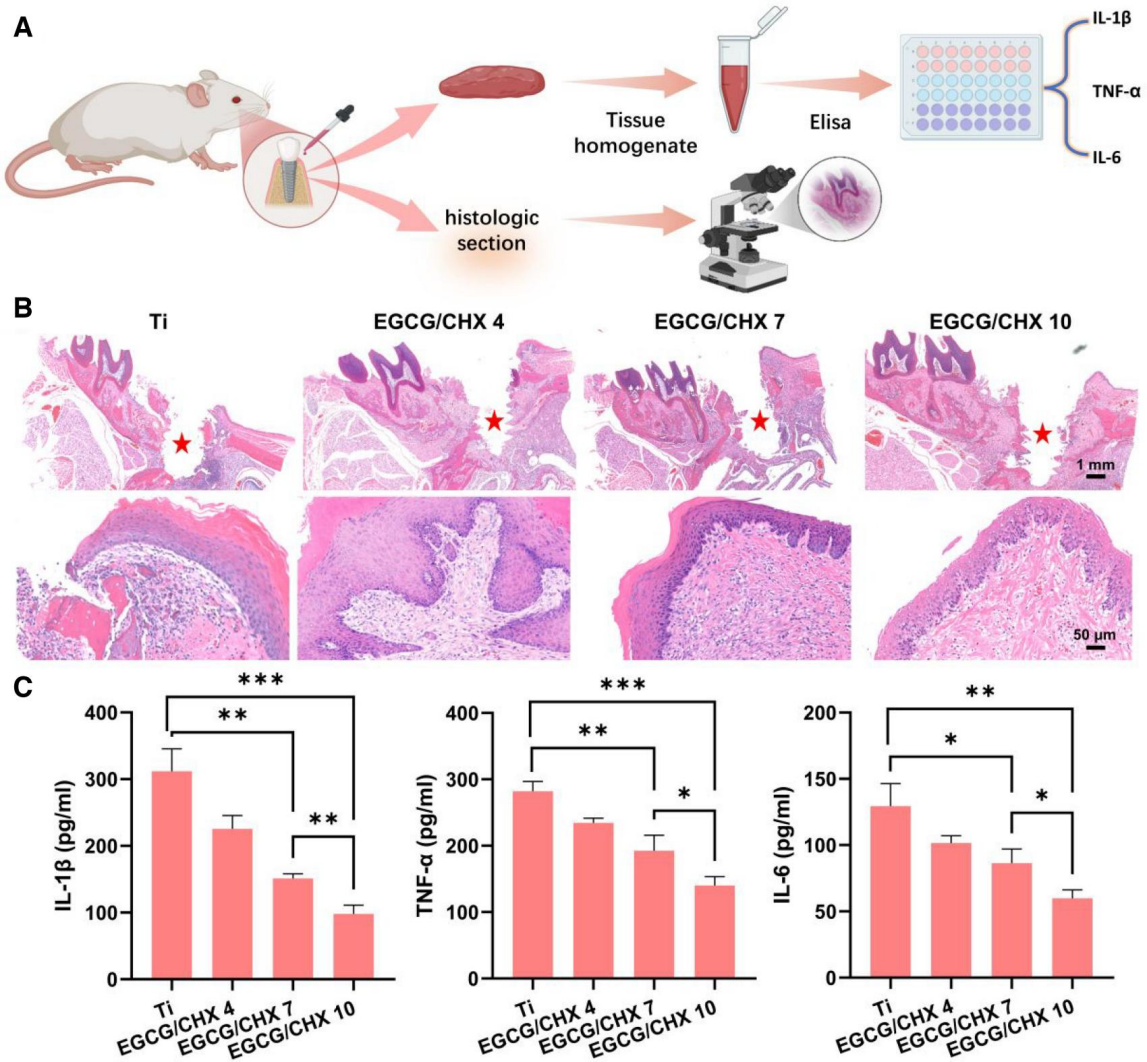
*In vivo* evaluation of dental implants in rats. (**A**) Implantation of dental implants in rats and the process of their performance evaluation; (**B**) HE staining of the periodontal tissues surrounding the implants in rats(“★”is the implant placement position); (**C**) quantitative analysis of inflammatory factors in the periodontal tissues surrounding the implants in rats.

To further evaluate the soft tissue sealing effect of the samples in the oral cavity, an intraoral implant model was established by implanting implants in the oral cavity of the rat (Figure 7A). The implantation process can compromise the transmucosal area of dental implants, making it susceptible to bacterial invasion. This vulnerability can trigger a severe pro-inflammatory M1 phenotype in macrophages and lead to excessive expression of pro-inflammatory cytokines, including TNF-α, IL-6 and interleukin-1β (IL-1β) [45]. These cytokines can affect the formation of STS and may result in peri-implant mucositis and the formation of deep periodontal pockets [46]. To evaluate the wound healing process and STS formation, low-magnification HE staining was employed. The tissue was divided into three distinct regions: peri-implant epithelium, implant contact area and subepithelial connective tissue surrounding the implant. In contrast, the coating groups (EGCG/CHX 4, EGCG/CHX 7 and EGCG/CHX 10) showed no obvious structural damage; however, mild inflammatory cell infiltration and relatively loose fiber arrangement were observed in the EGCG/CHX 4 and EGCG/CHX 7 groups. Compared to the Ti, EGCG/CHX 4 and EGCG/CHX 7 groups, the epithelial keratinization in the EGCG/CHX 10 group was both complete and continuous. The non-keratinized epithelium extended to the implant’s sealing area, establishing an adequate effective width. The contact and connective tissue regions were fully developed and densely packed. Importantly, no inflammatory infiltration was observed in the tissue, indicating that this group successfully established an effective soft tissue seal around the implant (Figure 7B). The invasion of bacteria triggered inflammation in the junctional epithelium, characterized by the formation of rete pegs and infiltration of a large number of neutrophils. This led to looser connections between epithelial cells [47]. As the duration of stimulation increased, the inflammation progressively worsened, ultimately causing detachment of the junctional epithelium. Consequently, in the pure Ti group, loose connective tissue with extensive infiltration of inflammatory cells was observed, and the tissue structure appeared more disorganized compared to other groups. However, due to the antibacterial effect of EGCG/CHX coatings, three EGCG/CHX-modified groups can form junctional epithelium with a good barrier effect, thereby resisting the further invasion of non-attached plaque and inhibiting the further development of inflammation. However, due to the long-term application of stimulation around the implant, there is still a stimulating effect on the peri-implant mucosa, resulting in different degrees of inflammation in the implant contact area and connective tissue area. The degree of inflammation around the implant depends on the anti-inflammatory effect of the coating. It is evident that the low inflammatory infiltration around the EGCG/CHX 10 implant, along with the complete and dense contact area and connective tissue area, demonstrate that this coating can more effectively achieve soft tissue healing and establish good STS in an environment with poor periodontal health.

Additionally, the quantification of inflammatory cytokines secreted by the tissue surrounding dental implants revealed that the levels of pro-inflammatory cytokines in the three EGCG/CHX groups were significantly lower than those in the Ti group. Notably, there was a decreasing trend in inflammatory factors as the pH increased (Figure 7C).These results indicate that EGCG/CHX-modified materials exhibit a pronounced inhibitory effect on initial inflammation induced by microorganisms when implanted *in vivo*. Previous studies have reported that lipopolysaccharides secreted by bacteria can stimulate macrophages and induce inflammatory responses; therefore, antimicrobial surfaces can alleviate inflammation and promote soft tissue healing and the STS formation by suppressing bacteria in the oral microenvironment [48]. This is consistent with *in vitro* cell/bacteria co-culture experiments that simulate the periodontal environment and macrophage inflammation assays in a bacterial environment, both of which demonstrate the good antibacterial and inflammation-inhibiting capabilities of the constructed surface.

**Figure 8.**
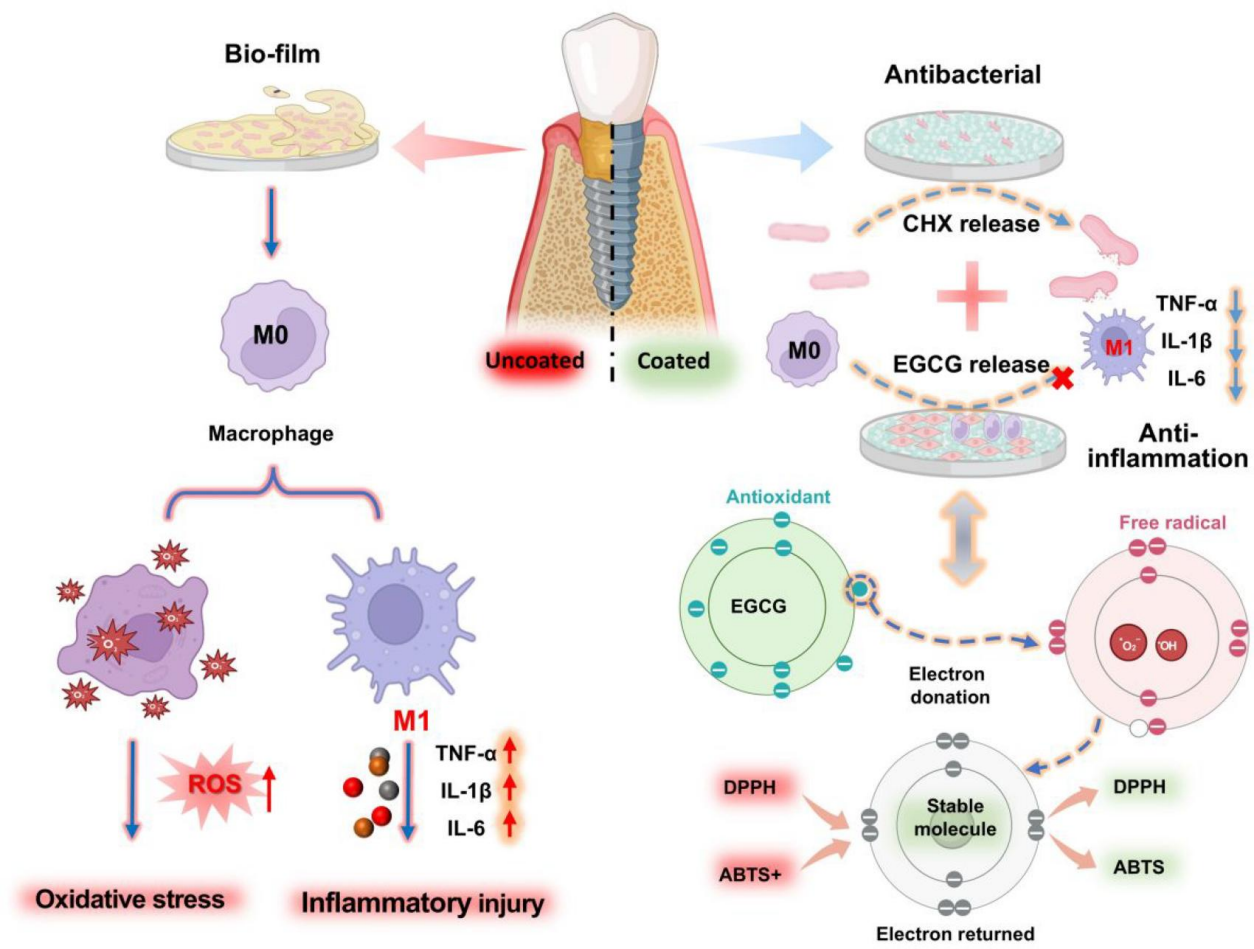
Scheme of design of EGCG/CHX coating to manage inflammation through antibacterial and antioxidation to boost STS though cell adherence promotion for the prevention and treatment of peri-implantitis.

STS around dental implants is critical for the long-term success of implant surgery, particularly in maintaining the health of the surrounding tissues, promoting healing, protecting the implant, facilitating soft and hard tissue integration [49]. The key factors influencing the STS include bacterial infection, local immune responses and cyto-compatibility of the implant surface [50]. Bacterial invasion is a primary factor contributing to inflammation [5]. An excessively activated immune response can disrupt the local cellular microenvironment through the release of immune factors and the recruitment of immune cells, as shown in Figure 8. Therefore, in addition to controlling bacterial infection, it is essential to modulate the immune response purposefully to effectively manage soft tissue encapsulation [51]. In this study, the commonly used clinical antibacterial agent CHX and the anti-inflammatory compound EGCG were selected to construct a coating that imparts antibacterial and anti-inflammatory properties to the implant surface. The loading and controlled release of CHX can effectively manage the initial antibacterial efficacy on the implant surface, ensuring the sterility of the local microenvironment. For inflammatory activation caused by bacterial infection and tissue damage, the sustained release of EGCG can mitigate such effects. EGCG achieves free radical scavenging by stabilizing electrons in free radicals, thereby reducing the activation of inflammatory cells, promoting the transformation of M1-type macrophages to M2-type macrophages, and simultaneously decreasing the release of pro-inflammatory factors, thus improving the immune microenvironment. Simultaneously, we examined the differences in coatings formed under varying reaction pH values. The results demonstrated that neutral and alkaline conditions were more favorable for coating formation, providing theoretical support for subsequent reactions between polyphenols and quaternary amine bases. In this study, the antibacterial efficacy of the coatings was validated through tests against various pathogenic bacteria. The immunomodulatory properties of the coatings were confirmed by analyzing immune cell phenotypes and pro-inflammatory factor expression. Additionally, the stability of the coatings is crucial for *in vivo* performance. We designed both *in vitro* and *in vivo* stability experiments, which proved that coatings formed under neutral and alkaline conditions exhibit superior stability. These coatings can maintain stable antibacterial and immunomodulatory effects for up to 14 days, thereby effectively supporting the formation of a complete STS on the implant surface.

Clinical transformation is the ultimate goal of scientific research, while biological safety serves as the foundational prerequisite for successful clinical translation. EGCG, the primary bioactive polyphenol in green tea, has been extensively documented for its anti-inflammatory, antitumor and antibacterial properties. The recommended daily intake of EGCG is 100–300 mg [52], while the concentration used in this study is considerably lower than this threshold, ensuring minimal biological toxicity. Similarly, chlorhexidine (CHX), a commonly employed clinical antiseptic in oral care, is considered safe at concentrations of 0.1–0.2% in mouth rinses [53]. The CHX concentration applied herein is substantially below this standard, further supporting its biosafety. Moreover, the interaction between EGCG and CHX is predominantly mediated by noncovalent bonding, preventing the formation of potentially toxic chemical structures. Collectively, the EGCG-based coating not only offers a versatile platform for surface functionalization but also ensures a high level of biological safety, making it highly suitable for clinical translation in implant surface modification.

## Conclusion

To address the biofunctionalization deficiency of implant abutments, an EGCG/CHX coating was successfully developed on titanium substrates. This innovation aims to facilitate the closure of peri-implant soft tissues and mitigate the incidence of peri-implantitis. The EGCG/CHX coating effectively prevents pathogenic bacterial invasion and reduces inflammatory responses in surrounding tissues through direct antibacterial action or sustained release of antimicrobial agents during the initial stages. Furthermore, as antimicrobial components are released, our findings indicate that the EGCG/CHX coating can create favorable conditions for tissue repair by modulating the immune microenvironment via a specific pathway. In summary, this study presents a multifunctional coating strategy that enhances soft tissue healing around implants, offering novel insights into the design of connective tissue barriers and immunomodulatory host defense systems for trans-mucosal implant components.

## Supplementary Material

rbaf046_Supplementary_Data
