# Supplementary material for: Construction of EGCG/chlorhexidine functionalized coating to reinforce the soft tissue seal at transmucosal region of implants
Source: Regen Biomater. 2025 May 20;12:rbaf046. doi: 10.1093/rb/rbaf046 (PMC12304417; doi:10.1093/rb/rbaf046)
Supplement: rbaf046_Supplementary_Data [file rbaf046_supplementary_data.docx]

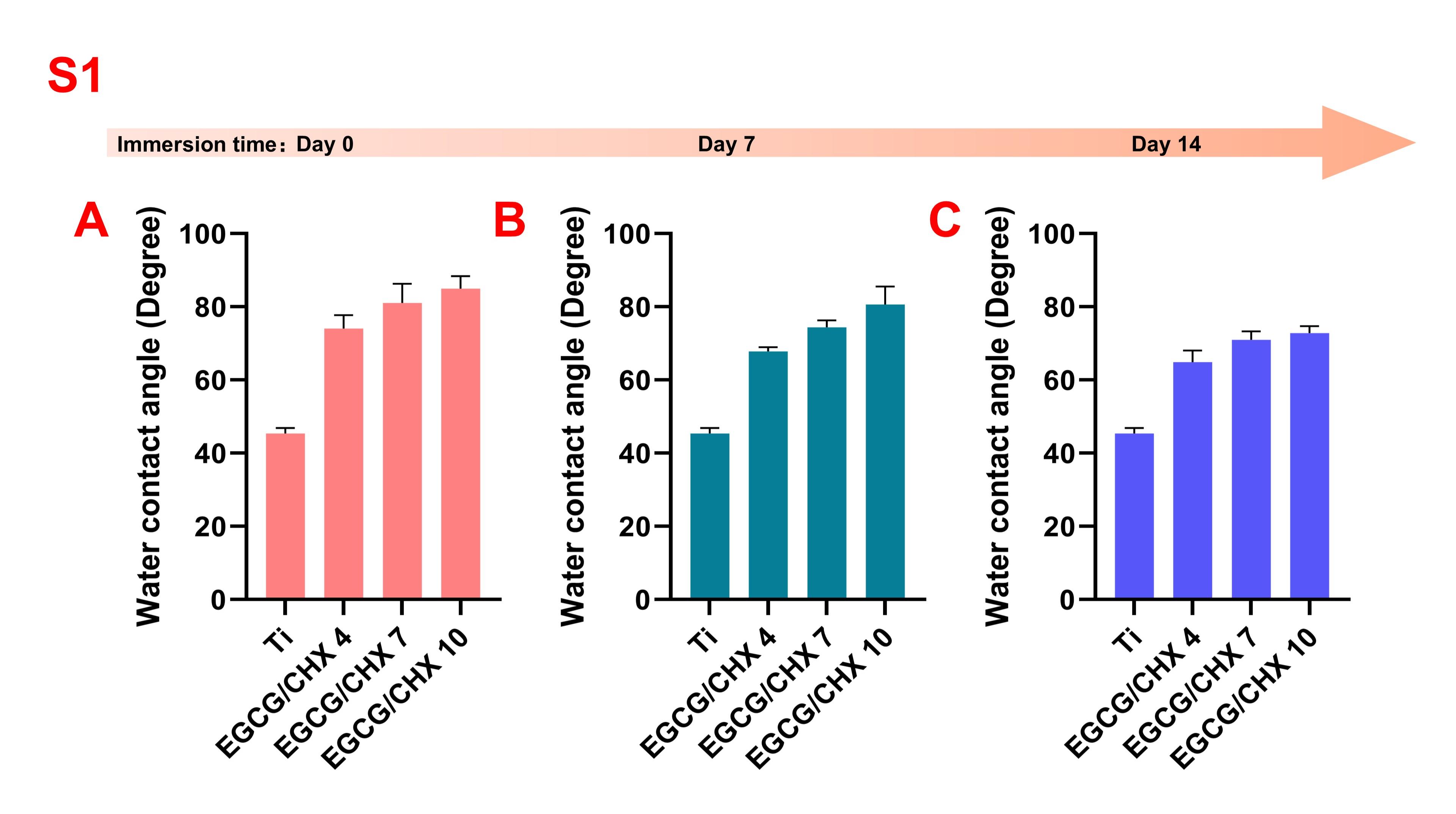


**S1** The water contact angle (WCA) at different pH levels before(A) and after sample release(B、C)





**S2** Measurement of the surface morphology of titanium.


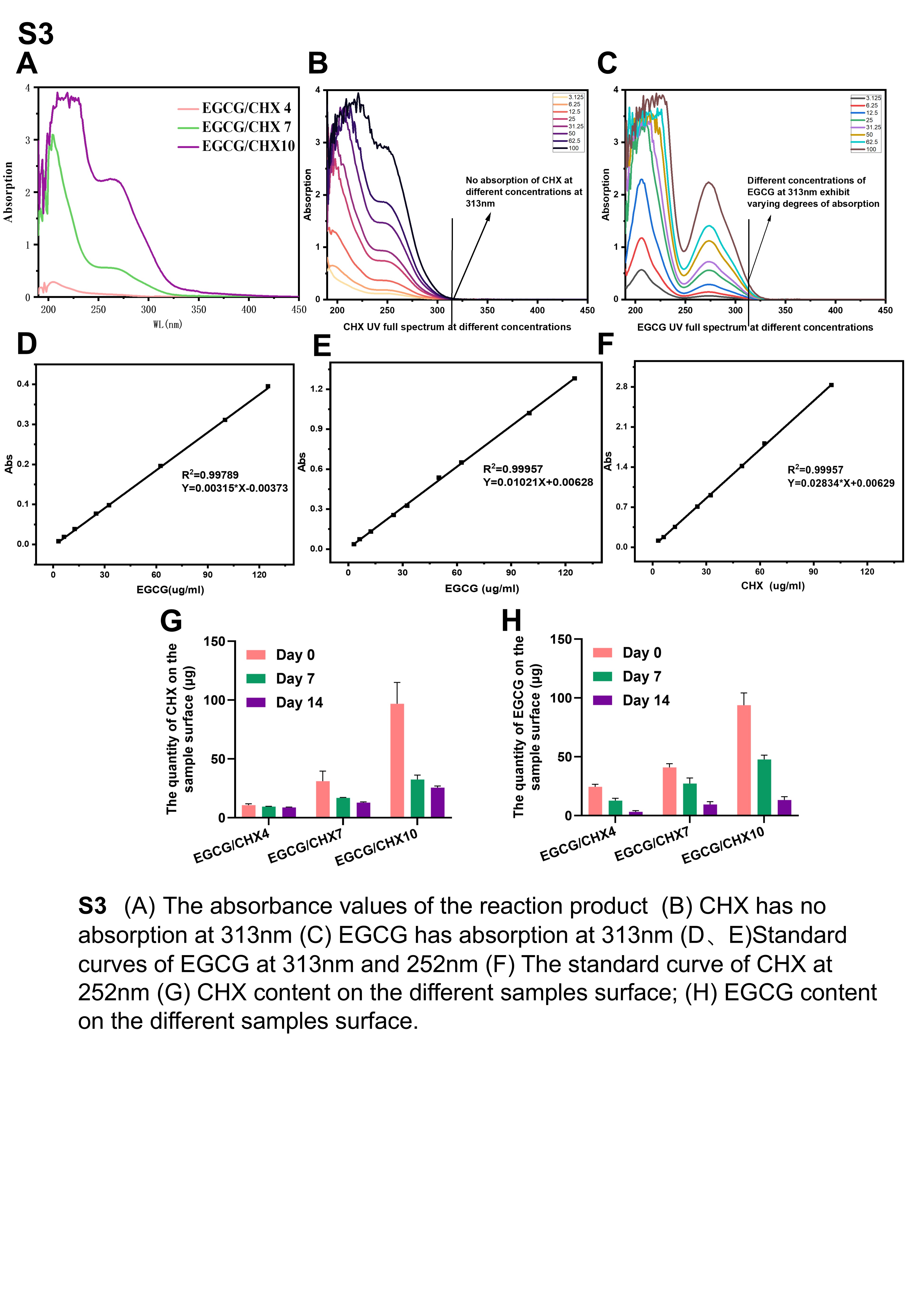


**S3** (A) The absorbance values of the reaction product (B) CHX has no absorption at 313nm (C) EGCG has absorption at 313nm (D、E)Standard curves of EGCG at 313nm and 252nm (F) The standard curve of CHX at 252nm (G) CHX content on the different samples surface; (H) EGCG content on the different samples surface.





**S4** The ζ-potential of the reaction products at different pH levels.


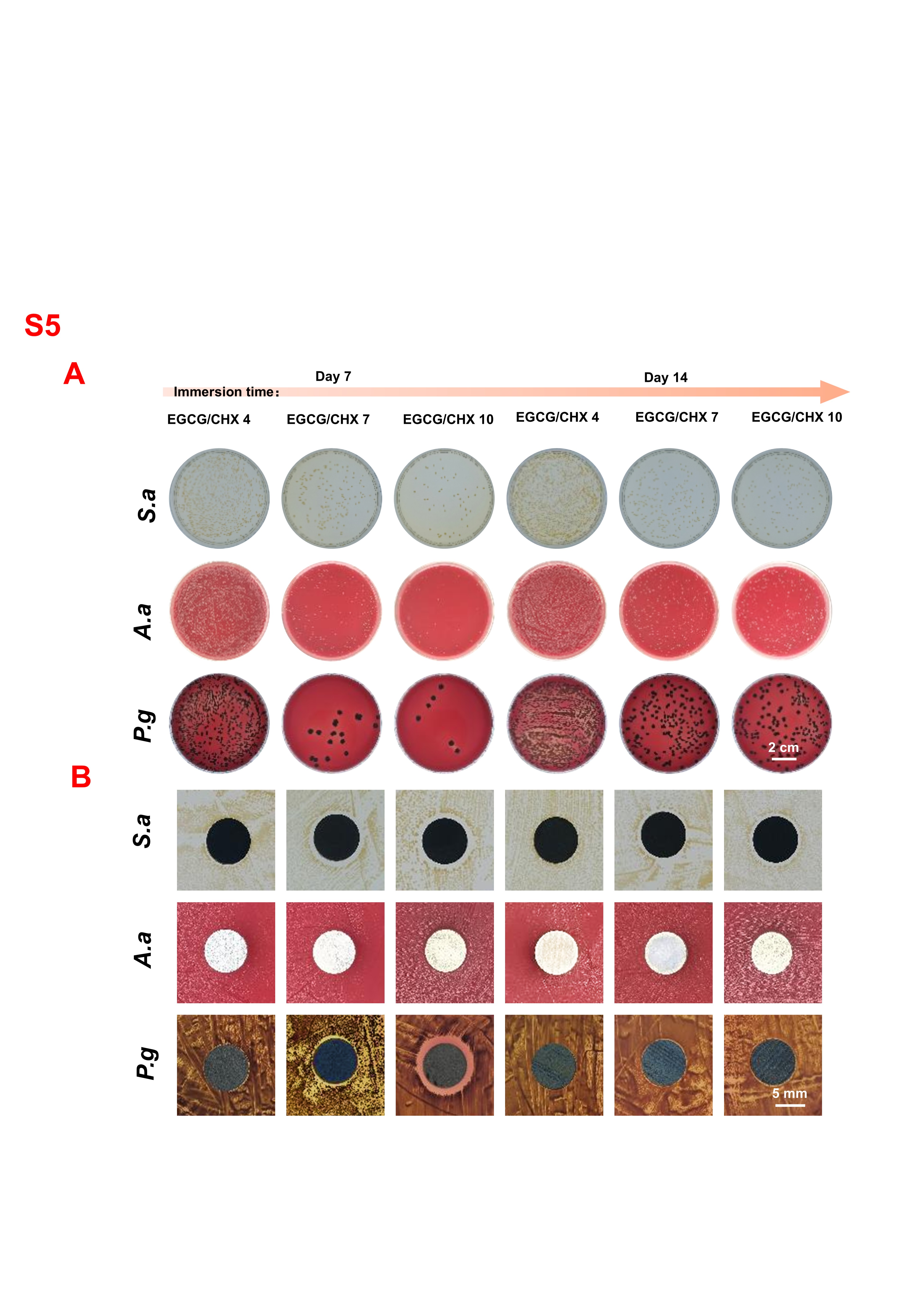


**S5** （A）Results of liquid antibacterial spread plate count before and after sample release;（B）zone of inhibition before and after sample release.
